# Development of a Brain Catheter for Optical Coherence Tomography in Advanced Cerebrovascular Diagnostics

**DOI:** 10.3390/bios15030170

**Published:** 2025-03-06

**Authors:** Tae-Mi Jung, Tahsin Nairuz, Chang-Hyun Kim, Jong-Ha Lee

**Affiliations:** 1 Department of Biomedical Engineering, Keimyung University, Daegu 42601, Republic of Korea; 1114436@stu.kmu.ac.kr (T.-M.J.); tahsin.bmb@nstu.edu.bd (T.N.); 2 Department of Neurosurgery, Keimyung University Dongsan Hospital, Daegu 42601, Republic of Korea

**Keywords:** optical coherence tomography, cerebrovascular OCT catheter, cardiovascular OCT catheter, cerebrovascular diagnostics

## Abstract

Optical coherence tomography (OCT) has been extensively utilized in cardiovascular diagnostics due to its high resolution, rapid imaging capabilities; however, its adaptation for cerebrovascular applications remains constrained by the narrow, tortuous anatomical structure of cerebral vessels. To address these limitations, this study introduces a cerebrovascular-specific OCT (bOCT) catheter, an advanced adaptation of the cardiovascular OCT (cOCT) catheter, with significant structural modifications for improved access to brain blood vessels. The bOCT catheter incorporates a braided wire within a braided tube, strategically reinforcing axial strength. The distal shaft was reconfigured as a single-lumen structure, facilitating unified movement of the rotating fiber optic core and guidewire, thereby reducing guidewire bending and augmenting force transmission stability. Additionally, the anterior protrusion was removed and replaced with a dual-lumen configuration, significantly enhancing lesion accessibility. The bOCT catheter’s performance was validated in a 3D physical model and an animal model, demonstrating pronounced enhancements in flexibility, pushability, and navigability. Notably, the pushability through curved flow paths significantly improved, enhancing access to cerebral blood vessels. Therefore, this innovation promises to revolutionize cerebrovascular diagnostics with high-resolution imaging suited to the complex brain vasculature, setting a new standard in intravascular imaging technology.

## 1. Introduction

With the growing adoption of endovascular interventions as the primary therapeutic approach for managing cerebrovascular pathologies—including brain aneurysms, ischemic occlusions, arterial dissections, and intracranial atherosclerotic disease—the necessity for high-precision imaging modalities has become paramount in facilitating optimal treatment planning and procedural guidance. While conventional imaging techniques remain instrumental in assessing arterial wall pathology, aneurysm morphology, neurovascular implant positioning, and post-procedural outcomes, their diagnostic utility is inherently constrained by technical limitations. Preclinical studies have established that precise apposition of neurovascular implants to the arterial wall, along with optimal coverage of the aneurysm neck, is strongly associated with enhanced aneurysm occlusion rates and improved patient outcomes [1,2,3]. This implies that inadequate implant positioning may necessitate additional interventions to achieve optimal occlusion. Moreover, a more comprehensive evaluation of arterial wall pathology, including residual thrombi and the identification of the key anatomical landmarks such as perforating arteries, can significantly inform device selection and placement in endovascular stroke treatments [4]. However, current noninvasive imaging modalities often fall short due to limitations in spatial resolution, contrast, and susceptibility to metal-induced artifacts, rendering accurate assessments challenging [5,6]. Although intravascular (IV) imaging methods have demonstrated efficacy in the imaging of coronary and peripheral arteries, they are not yet suitable for routine cerebrovascular applications.

Over the past decade, intravascular optical coherence tomography (OCT), a catheter- based noninvasive imaging modality, has garnered substantial recognition for its exceptional capability to capture high-resolution cross-sectional images of tissues in blood vessels. This advanced technique achieves unparalleled imaging precision by measuring the amplitude of the backscattered light (light echo or reflectance) reflected from a near-infrared or infrared light source directed toward the vessel wall. OCT has been extensively leveraged in coronary imaging, facilitating the precise delineation of lumen morphology and comprehensive quantification of coronary artery disease severity [7,8] in addition to playing a critical role in the detailed assessment of emerging generations of intracoronary stent devices [9,10]. Beyond its established role in cardiovascular interventions, the potential for intravascular OCT to revolutionize the diagnostic and therapeutic paradigms of cerebrovascular pathologies has attracted significant scholarly interest [11,12].

Nevertheless, currently available OCT catheters are deficient in the requisite flexibility needed to traverse the highly tortuous and convoluted architecture of cerebrovascular vessels, thus rendering them unsuitable for imaging within regions of high tortuosity and incompatible with the complexities inherent in the neurovascular clinical workflow [11]. The widely used cOCT catheters, such as Dragonfly™ (Abbott) and FastView™ (Terumo) [13], provide high-resolution (2–10 µm) imaging but are limited in their ability to access intricate neurovascular regions, particularly due to the 2 cm tip protrusion ahead of the OCT sensor. Consequently, the clinical utilization of IV imaging has been largely confined to the posterior cerebral circulation in a limited subset of patients exhibiting relatively mild vascular tortuosity [14], as well as to proximal and straight segments of the internal carotid arteries [15,16]. Furthermore, a critical limitation of the current imaging systems lies in their constrained field of view, which significantly hampers their capacity to provide a comprehensive characterization of larger and more intricate carotid arteries, especially those with diameters exceeding 5 mm, as well as the detailed evaluation of intracranial aneurysms [17]. This insufficiency underscores the need for technological advancements to expand the applicability and efficacy of IV imaging within cerebrovascular diagnostics. While intravascular ultrasound (IVUS) catheters, such as Eagle Eye Platinum (Philips Volcano) [18], provide deeper tissue penetration, their lower resolution (100–150 µm) makes them inadequate for detailed visualization of fine vascular structures, thrombus morphology, and neurovascular stent apposition, further underscoring the demand for specialized OCT solutions designed specifically for cerebrovascular imaging and interventions.

In this study, a specialized OCT catheter was developed to address the unique challenges of cerebrovascular imaging, involving the strategic adaptation and refinement of a conventional cOCT catheter into a cerebrovascular-specific bOCT catheter. The design process focused on the precise control of the insertion and forward movement of a guidewire and an imaging catheter. The resulting bOCT catheter features improved flexibility and adaptability, enabling it to traverse even the highly tortuous cerebrovascular vessels, thereby providing enhanced access to lesion sites for more precise diagnostics and interventions.

## 2. Materials and Methods

### 2.1. Study Overview

This study conceptualized the bOCT catheter as an adaptation of the cOCT catheter (Dragonfly, Image Optical Coherence Tomography Guide Catheter, Abbott) to overcome the navigational constraints imposed by the narrow, tortuous cerebral blood vessels. A prototype bOCT catheter was meticulously designed and fabricated for cerebrovascular applications, with its usability rigorously confirmed in a three-dimensional (3D) physical model and an animal model. The catheter’s design evolution was predicated on addressing the inherent limitations of conventional cOCT catheters, emphasizing key structural enhancements. (1) To increase the contact surface between the guidewire and the microcatheter and augment pushability, the over-the-microwire segment—extending from the distal entry port to the proximal exit port—was elongated to approximately 300 mm, significantly exceeding the 20 mm span of coronary IV-OCT. (2) The guidewire lumen was structurally integrated with the OCT lens optical fiber cable, eliminating discontinuities that could impede force propagation. Furthermore, the microcatheter composition incorporated a mixed braided–non-braided structure, wherein the distal 30 mm remained non-braided to prevent interference from braided coiling, while the proximal 270 mm featured a reinforced braided configuration to improve pushability. This braided proximal shaft significantly enhances axial strength compared to the extruded catheter and improves the pushability performance required when entering the flow path and has the advantage of bending less during the procedure.

Additionally, the distal shaft was designed as a single-lumen tube to unify the moving path of the rotating fiber optic core and the guidewire, providing a flexible connection between the proximal and distal shafts. The catheter’s shaft is made from three different polymer materials (Pebax^®^ 4033, 5533, and 7233, Arkema, Colombes, France), with rigidity gradually decreasing from the proximal to distal sections, allowing for enhanced flexibility in the distal part of the catheter. The design also focuses on reducing the flexibility of the lower part of the microwire/guidewire while increasing the force to support the catheter tube. Additionally, the design improves upon the cOCT catheter by replacing the front protrusion with a dual-lumen configuration. This allows one lumen to carry the light source and the optical cable, while the other guides the microwire, which exits from the side further back from the tip, improving catheter accessibility. The changes are compared with the cardiovascular OCT catheter in Table 1.

**Figure 1:**
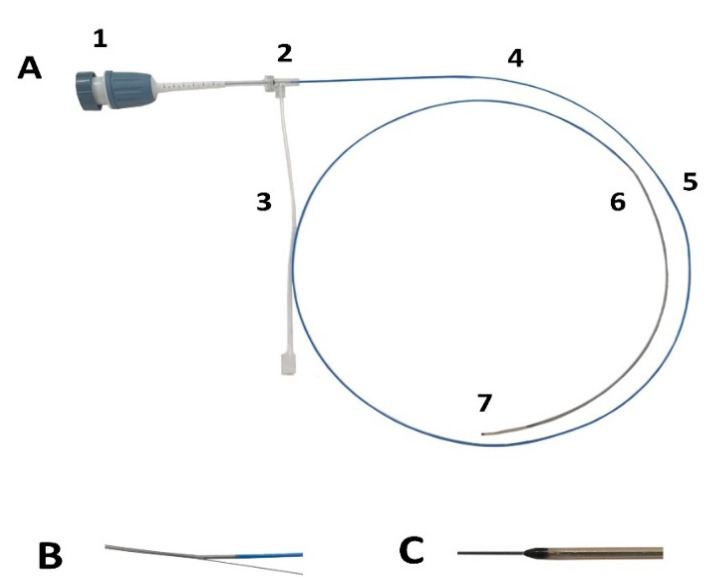
Prototype bOCT catheter. (**A**) Whole catheter; (1) PIU (connector), (2) side-arm luer, (3) manifold, (4) proximal shaft (braided tube), (5) distal shaft (braided tube), (6) tip, (7) guidewire port/purge exit. (**B**) Guidewire through the proximal exit port on the side. (**C**) Guidewire through the entry port at the distal end.

The prototype bOCT catheter was developed according to the abovementioned specifications, as shown in Figure 1, and the descriptions of each part of the OCT catheter are provided in Table 2.

### 2.2. Structural Design of the bOCT Catheter for Cerebrovascular Diagnostics

**Figure 2:**
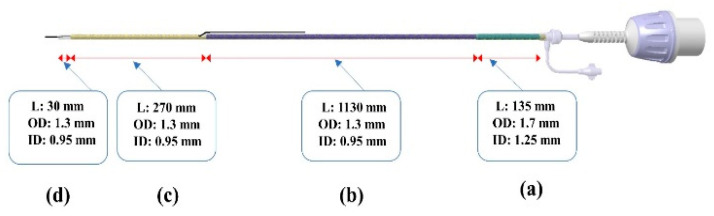
Schematic diagram of the segment of the bOCT catheter including the outer diameter (OD) and the inner diameter (ID). (**a**) Proximal braided shaft part (135 mm). (**b**) Proximal braided part (1130 mm). (**c**) Distal braided shaft part (270 mm). (**d**) Distal non-braided OCT lens part (30 mm).

The catheter developed for this study comprises a shaft and a manifold handle, with the shaft divided into four distinct segments, from the proximal to distal parts. The structure of the catheter with the inner and outer diameters of each layer is depicted in Figure 2.

The first and second segments of the shaft, which are the proximal parts, consist of a three-layered braided structure. To enhance the overall rigidity of the proximal part, an elastomer material outer jacket (Pebax 7233 SA 01 MED) with a Shore hardness of 70D was extruded. The middle layer features 16 flat wires (SUS304V, 0.01 × 0.05 mm, Ulbrich, Inc., North Haven, CT, USA), intricately braided in a herringbone pattern with a density of 45 picks per inch (PPI) to enhance the catheter’s pushability while minimizing kinking. The innermost layer comprises a polytetrafluoroethylene (PTFE) liner (from ZEUS, Inc., San Jose, CA, USA), which have a low coefficient of friction, ensuring stable transport of the coil equipped with an optical mirror. To fabricate the braided shaft, the outer jacket, the braiding wire, and the polytetrafluoroethylene (PTFE) liner of the proximal part underwent vertical lamination (Machine Solutions, Inc., Flagstaff, AZ, USA) through the reflow process at 200 °C and 1.0 mm/s. The processing methods for each part of the bOCT catheter are mentioned in Table 3, and the descriptions of these processing methods are provided in Supplementary Data S1.

In the third segment of the shaft, the distal part, a single material (Pebax 5533 SA 01 MED, Foster, Putnam, CT, USA) is used to enhance trackability within the microchannel, minimize guidewire bending, and increase the tube’s structural strength. Toward the catheter tip, hardness gradually decreases, which aids in flexibility. The distal shaft includes both braided and non-braided sections to optimize performance: the braided tube design is used to reduce rotation, while the non-braided section contributes to improved flexibility and pushability.

The 4th segment, the distal tip part, uses a biocompatible medical polymer (Pebax 4033 SA 01 MED) with a Shore hardness of 40D to prevent potential damage to blood vessels. The tapered tip was manufactured through a tip-forming process using an electromagnetic high-frequency induction heating method. During this process, a polymer ring blended with Pebax and tungsten (Pebax:tungsten = 2:8 wt. %) was formed and mounted at the tip, providing radiopaque functionality.

**Figure 3:**
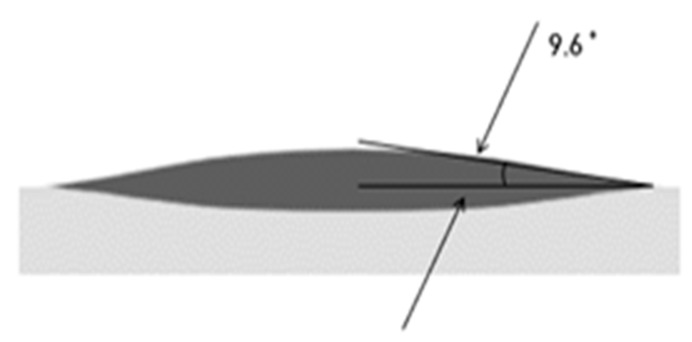
Surface contact angle after hydrophilic coating.

Moreover, to enhance the catheter’s functional efficacy during vascular procedures, a hydrophilic coating was applied to the catheter shaft, significantly reducing friction and increasing its pushability. Given that blood consists of approximately 55% plasma, of which 90% is water, when a hydrophilic coating is applied in conjunction with saline solution to the shaft surface, the friction between the shaft and blood is significantly lowered upon entering the blood vessel. This reduction in friction greatly improves the catheter’s pushability. Figure 3 illustrates the lowered surface contact angle after hydrophilic coating, indicating enhanced hydrophilicity.

The hydrophilic coating was achieved through a dip-coating process using a hyaluronic acid (HA)-based solution (Noacoat_p, Noanix, Republic of Korea), followed by a heat-curing process. The hydrophilic coating was performed as follows:

(1) The catheter shaft was first cleaned with a solution of isopropyl alcohol (IPA, Sigma-Aldrich, St. Louis, MO, USA) and deionized water (D.I. water) in a 1:1 ratio.
(2) The shaft was then air-dried for 10 min at room temperature.
(3) Dip coating was performed by immersing the shaft in a base coating solution (Noacoat_base, Noanix, Republic of Korea) and lifting it slowly.
(4) The base-coated shaft was dried in an oven at 60 °C for 10 min, then air-dried at room temperature for an additional 10 min.
(5) The catheter shaft was re-immersed in a top coating solution (Noacoat_p, Noanix, Republic of Korea).
(6) Finally, the coated shaft was heat-cured by drying it in an oven at 60 °C for 120 min.

### 2.3. Evaluation of the Catheter’s Mechanical Performance

The mechanical performance of the bOCT catheter was evaluated and compared with an existing cOCT catheter using the ASTM F2394 standard simulated blood vessel flow path [19]. Trackability and pushability were selected as the key performance parameters due to their relevance in cerebral vascular applications. Trackability was defined as the resistance encountered by the catheter as it traverses through the vasculature, while pushability was defined as the extent to which the force applied to the proximal end of the catheter is transmitted to the distal tip. This metric allows for the assessment of the catheter’s entry performance within the tortuous vascular pathways. For quantitative analysis, real-time force measurements were taken with a force sensor attached to the motor, which was then graphed.

To replicate vascular conditions, distilled water was used in the flow path instead of blood, and a 0.014-inch guidewire was employed to simulate catheter passage. The travel distance was set at 240 mm from the inlet to allow for a thorough investigation of potential catheter kinking. When kinking occurred, the distance traveled and the resistance force were recorded for analysis (details about the catheter trackability measuring device are provided in Supplementary Data S2).

### 2.4. Confirmation of Catheter Usability via a Physical Model

In this study, based on DICOM files acquired from patients’ CT data, their cerebrovascular vessels were modeled through segmentation and 3D reconstruction using Mimics (Materialize, Louvain, Belgium), a 3D medical image processing software. After the patient-specific 3D modeling of cerebrovascular blood vessels, the prototype model was printed using Raise3D Pro2 Plus (Raise3D, Korea), a 3D printer based on the fused deposition modeling method. The printed prototype model was made of ABS (acrylonitrile butadiene styrene), chosen specifically for its compatibility with the acetone dissolution process required later in the procedure. Following the initial printing, the ABS-based cerebrovascular prototype was dipped in silicone (KE-1606, Shimadzu, Tokyo, Japan) to form a durable silicone layer. A patient-specific cerebrovascular prototype model, printed using a 3D printer, was dipped in silicon (KE-1606, Shimadzu). To ensure minimal bubble formation during the silicone coating process, the dipping, defoaming, and initial curing steps were repeated at least five times. After the final silicone dipping, the model was cured in an oven at 60 °C for 24 h to allow the silicone to fully set and then immersed in acetone to dissolve the internal ABS prototype. This process resulted in a patient-specific, silicone-based physical model of the cerebrovascular structure.

### 2.5. Confirmation of Catheter Usability Through an Animal Model

A pig weighing 25–30 kg was used to evaluate the usability of the catheter. For anesthesia, a combination of 30 mg/kg Rompun (20 mg xylazine) at 0.15 mL/kg, 0.5 mg atropine, and Zoletil 50 (125 mg tiletamine, 125 mg zolazepam) was injected intramuscularly. An injection line was maintained in the pig’s ear vein, and during the catheterization procedure, a 2 mL dose of a 3:1 solution of Rompun and Zoletil 50 was injected at intervals of 30 min to 1 h as needed. During the catheterization, a guiding catheter was inserted sequentially through the femoral artery, the abdominal–thoracic aorta, the common carotid artery of the aortic arch, and into the proximal external carotid artery. Both the bOCT and cOCT catheters were then inserted through the curved external carotid artery to assess catheter movement. All procedures were performed in compliance with the Guiding Principles in the Care and Use of Animals: National Research Council, 1996, and the Animal Welfare Committee of the Daegu-Gyeongbuk Medical Innovation Foundation: approval number DGMIF-20012201-01.

## 3. Results

### 3.1. Measurement of the Mechanical Performance of the cOCT and bOCT Catheters

**Figure 4:**
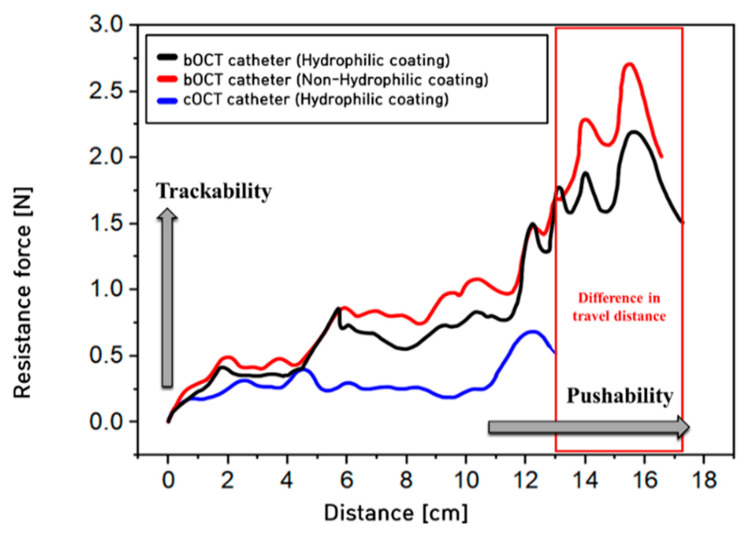
Graph showing the trackability and pushability performance in the vascular simulated flow path according to the catheter specifications.

The developed bOCT catheter was fabricated as a braided tube to pass through a curved channel; thus, the resistance when entering the simulated blood vessel flow path was slightly increased. This initial increase in resistance is compensated by a significantly enhanced performance within the curved channel, where the bOCT catheter successfully penetrated the simulated blood vessel flow path that the cOCT catheter could not due to issues with kinking. Figure 4 illustrates the pushability and trackability results of the bOCT catheter in comparison with the cOCT catheter in the standard simulated blood vessel flow path. The pushability results in this study confirmed the superior performance of the proposed braided shaft structure of the bOCT catheter compared with the conventional cOCT catheter. The cOCT catheter could penetrate up to 13 cm in the standard simulated blood vessel flow path, whereas the bOCT catheter demonstrated an increased penetration capability, reaching beyond 17 cm, indicating an approximately 30% improvement with the hydrophilic coating. Moreover, in the comparison of trackability between the two catheters, the maximum resistance measured within the range of distances traversed by both catheters in the standard flow path was analyzed. The cOCT catheter exhibited a measured resistance of around 0.7 N, while the bOCT catheter showed a greater resistance of almost 1.5 N. This result suggested that the bOCT catheter’s design, featuring a shortened non-braided section and a lengthened braided section, allowed it to pass through the curved flow path without kinking, thus significantly improving its usability in complex vascular routes.

These performance differences are closely related to the flexural rigidity, column strength, and contact area of the braided wire in the bOCT catheter. When catheters with braided wire are used, they exhibit increased resistance within the simulated blood flow path due to high flexural stiffness and column strength. Additionally, the proximal force was efficiently transmitted through the contact areas between the braided wires of the catheter; therefore, the catheter could enter flow paths with a small curvature radius.

As shown in Figure 4, trackability was measured based on the hydrophilic coating on the surface of the catheter, indicating that the hydrophilic coating significantly enhances trackability by reducing surface friction. The hydrophilic coating on the bOCT catheter results in smoother penetration through the flow path, lowering resistance and improving the overall performance compared to the non-hydrophilic version.

### 3.2. Validation of the bOCT Catheter Using a Physical Model

**Figure 5:**
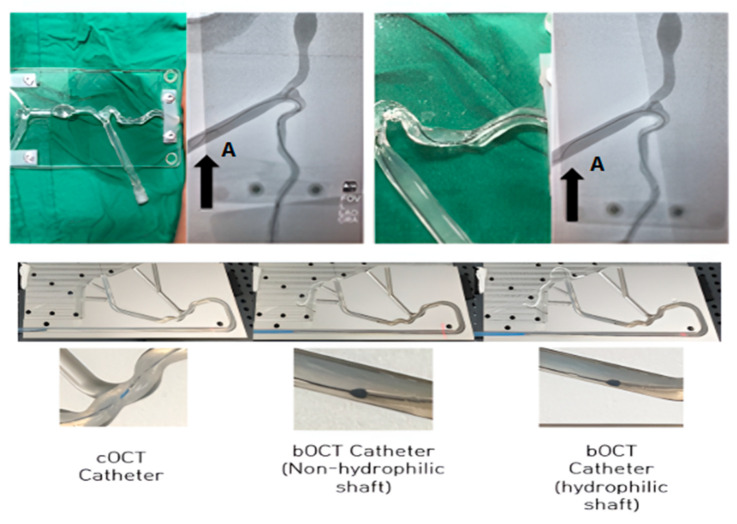
Simulated blood vessel flow path (ASTM F2394) for investigating the mechanical performance of the cOCT and bOCT catheters. Distilled water was placed in the flow path instead of blood, and a 0.014-inch guidewire was used to analyze mechanical performance. The travel distance was set to 240 mm from the inlet to sufficiently observe catheter kinking. Kinking occurred at the enlarged flow point in the photo, and the maximum drag force at this point was measured.

The trackability analysis results using a physical model was also similar to the results in the previous section. When a clinician tested the cOCT and bOCT catheters, as shown in Figure 5, the conventional cOCT catheter could not pass through a tortuous flow path and buckled at point A (indicated with an arrow in Figure 5). Conversely, the bOCT catheter exhibited a stable entry performance because it effectively transmitted the force from the proximal part. In addition, it passed point A and a blood vessel branch bent at 90°. The bOCT catheter with a braided wire exhibited superior trackability performance compared with the existing cOCT catheter.

### 3.3. Validation of the bOCT Catheter Using an Animal Model

To verify the usability of the developed brain catheter, comparisons were made between an intermediate catheter, the conventional cOCT catheter, and the newly manufactured bOCT catheter in terms of entry performance in the external carotid artery. The procedures included femoral artery sheath insertion, guiding catheter placement, and common carotid artery angiography. Specifically, the study focused on analyzing the degree of elevation while passing through the curved part of the external carotid artery and the degree of pressure or tension when ascending.

**Figure 6:**
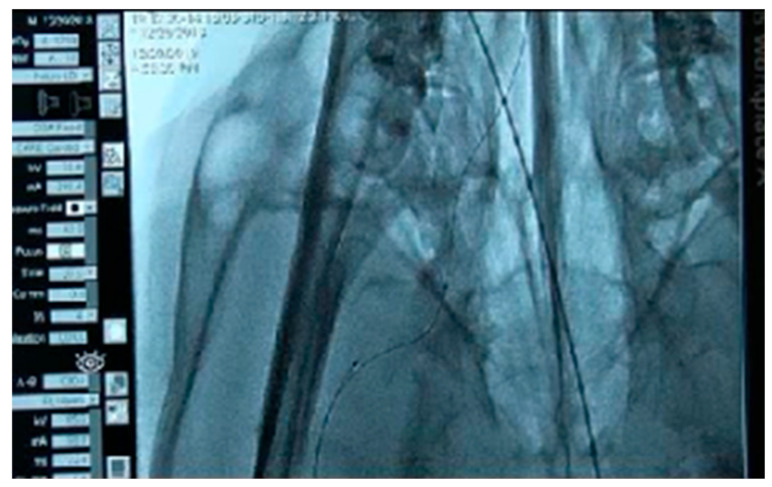
Insertion of the cOCT into the right external carotid artery. The catheter moves upward as it passes through a tortuous site.

**Figure 7:**
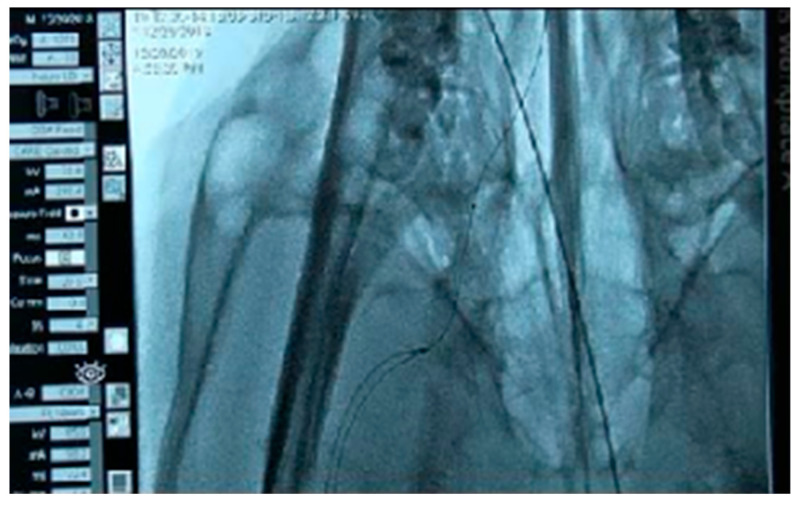
Insertion of the cOCT catheter into the right external carotid artery. The catheter no longer enters when tortuosity is exceeded.

**Figure 8:**
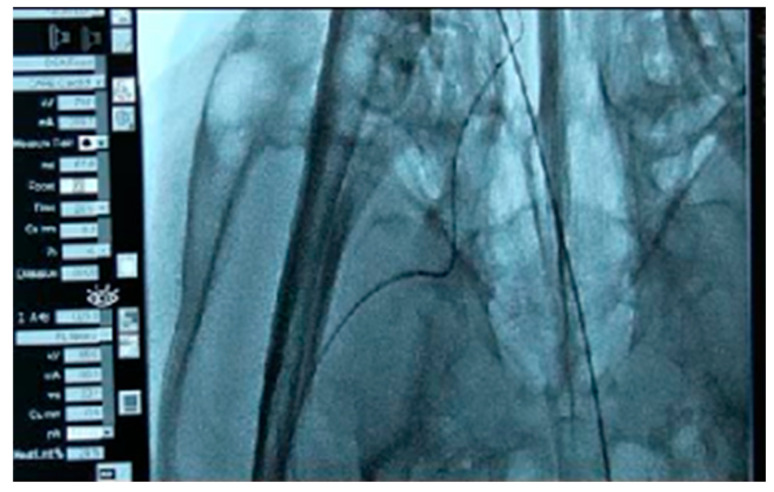
Insertion of the bOCT catheter into the right external carotid artery: good entry without excessive tension when passing through two areas of tortuosity.

The analysis of Figure 6, Figure 7 and Figure 8 demonstrates the differences in performance between the cOCT and bOCT catheters when inserted into the right external carotid artery. In Figure 6, the cOCT catheter is raised to a certain level.

However, when the catheter reached a tortuous section as shown in Figure 7, it opened and stopped progressing further.

In contrast, when the bOCT catheter was inserted into the right external carotid artery (Figure 8), the catheter encountered less tension, allowing it to successfully enter and pass through two tortuous sections.

Therefore, this animal study also confirmed that the cOCT catheter cannot pass through the curvatures of cerebral blood vessels, whereas the bOCT catheter can pass successfully 3–4 bends in curved areas without severe tension.

## 4. Discussion

Cerebrovascular diseases, such as ischemic stroke, aneurysms, and arterial stenosis, represent complex clinical challenges due to the intricate anatomy and physiological demands of the cerebral vasculature [20]. OCT, with its high-resolution (2–10 μm) cross-sectional imaging, captures light reflectance from vessel walls, revealing critical details about vascular wall pathology [21]. Although OCT has been firmly established as a gold-standard imaging modality in cardiology providing high-resolution intravascular visualization for coronary diagnostics, conventional coronary OCT catheters are inherently constrained by limited structural adaptability and inadequate flexibility, rendering them unsuitable for navigating the highly tortuous cerebral vasculature. These catheters face significant challenges in traversing sequential vascular curvatures, further exacerbated by a 2 cm tip protrusion, which restricts their flexibility in these narrower, more tortuous pathways. To address these limitations, in this study, we developed a modified OCT catheter specifically designed for the brain, adapted from the conventional coronary model, with key structural refinements to enhance vascular accessibility and procedural efficiency. The modifications include removing the tip protrusion and adopting a dual-lumen structure to extend the catheter’s reach and navigability within cerebral vessels. Additionally, a hole was created for the light source and the optical cable, while a separate opening was designed for the microwire, allowing it to exit from the side rather than the tip, thereby enhancing the catheter’s flexibility, trackability, and intravascular adaptability. Furthermore, the catheter’s braided structure, dual-lumen configuration, and optimized optical fiber alignment were specifically designed to ensure stable light transmission and minimal signal distortion, maintaining imaging integrity throughout the procedure.

This study advances a cutting-edge paradigm by conceptualizing and rigorously assessing a bOCT catheter optimized for navigational precision and diagnostic efficacy within the intricate cerebrovascular network. Both the conventional cOCT catheter and the newly developed bOCT catheter were evaluated for their ability to pass through the curved sections of the external carotid artery using a 3D physical model where clinicians assessed the degree of movement, pressure, and tension required for each catheter. Due to inherent structural limitations, the cOCT catheter, designed for the relatively straight pathways of coronary arteries, is unsuitable for cerebral vessels that contain multiple sharp curves. Besides 3D modeling, we also confirmed that the cOCT catheter could not reliably traverse these complex curvatures through testing with an animal model. In contrast, the bOCT catheter proved capable of traversing 3–4 consecutive bends with minimal resistance and without causing excessive tension. Interestingly, preliminary observations during usability testing revealed no noticeable degradation in image resolution or clarity, indicating that the structural modifications of the bOCT catheter also effectively preserve high-fidelity OCT visualization under operational conditions. This breakthrough represents a crucial development in the OCT technology, opening possibilities for more precise imaging and diagnostics within the complex cerebrovascular anatomy.

Moreover, the performance of the bOCT catheter demonstrates significant advantages over the existing neurovascular imaging modalities, particularly in spatial resolution, vessel wall visualization, and procedural guidance. Compared to digital subtraction angiography (DSA), the gold standard for neurovascular imaging [22], the bOCT catheter provides real-time, high-resolution cross-sectional imaging of vascular walls and thrombus composition, whereas DSA primarily offers lumen-based contrast imaging with lower structural detail. Computed tomography angiography (CTA) and magnetic resonance angiography (MRA), while noninvasive, suffer from lower spatial resolution, metal-induced artifacts, and limited capability for guiding endovascular procedures [23], whereas the bOCT catheter enables detailed assessment of the vascular pathology with precise lesion characterization. Compared to intravascular ultrasound (IVUS) which has a much lower spatial resolution (100–150 µm) [24], the bOCT catheter enables superior visualization and identification of the microvascular pathology.

Notably, these findings underscore the proposed bOCT catheter’s substantial promise for advancing cerebrovascular diagnostics and interventions across diverse clinical applications. It can traverse vessels with 3–5 curves to reach lesions, enabling the identification of cerebrovascular stenosis and acute occlusions. Using 3D reconstruction, the catheter can precisely reveal the shape and composition of blood clots (distinguishing between red and white thrombi) and detect arteriosclerosis, detailing its structure and composition. Additionally, the bOCT catheter can locate the origin of small perforating arteries entering the brain at stenosis sites, measure balloon diameters accurately during balloon angioplasty, and determine the exact length and diameter of stents. It is also useful for examining blood vessels inside a stent post-insertion and assessing the composition of cerebral aneurysm walls, offering valuable insights for treatment purposes.

However, this study has certain limitations, particularly emphasizing the development of a bOCT catheter aimed at enhancing trackability and pushability to traverse tortuous vessels. As a result, comprehensive evaluation of the catheter’s function was not prioritized. Moreover, the bOCT catheter was tested mainly in controlled and animal models, which may not entirely reflect the complexity and variability of human cerebrovascular structures, potentially affecting its applicability in clinical settings. Furthermore, the catheter’s long-term durability and performance under continuous use remain unverified. Future research will focus on enhancing the clinical feasibility of the bOCT catheter through biocompatibility assessments, accelerated aging studies, and preclinical trials to evaluate its long-term usability, thrombotic response, and structural integrity. To further validate its mechanical advantages, additional axial load-bearing tests will provide deeper insights into its mechanical durability. Future studies will further examine the catheter’s usability by incorporating a broader range of preclinical models, including larger animal studies with complex vascular structures, to comprehensively evaluate trackability, pushability, and imaging precision across varied cerebrovascular environments. Additionally, comparative analyses with existing neurovascular devices will aid in refining its design, functional optimization, and clinical applicability. The catheter’s impact on blood flow dynamics and vessel wall interactions will also be investigated through computational fluid dynamics (CFD) simulations and in vivo imaging studies, assessing shear stress alterations, hemodynamic flow patterns, and endothelial responses to ensure minimal vascular trauma during navigation. Furthermore, a quantitative evaluation of the OCT image quality, including the signal-to-noise ratio, axial resolution, and imaging consistency, will be conducted using phantom and in vivo imaging experiments to validate its diagnostic performance in different vascular conditions. Collectively, these efforts will refine the optimization and broader applicability of the bOCT catheter for advanced neurovascular interventions.

## 5. Conclusions

The development of a brain-specific OCT catheter marks a significant technological breakthrough in medical imaging and interventional strategies for cerebrovascular diseases. Conventional cOCT catheters, while effective in cardiovascular applications, are inherently constrained in their ability to navigate the intricate, sharply curved, and structurally complex architecture of the cerebral vasculature. This study paves the way for high-resolution imaging within cerebral vessels by developing an optimized bOCT catheter, adapted from the cOCT technology, enabling accurate diagnosis and characterization of cerebrovascular stenosis, acute occlusions, and aneurysms. The enhanced flexibility and structural refinements of the bOCT catheter facilitate superior diagnostic accuracy, targeted therapeutic interventions, and reduced procedural risks, ultimately advancing clinical outcomes in cerebrovascular disease management.

## Figures and Tables

**Table 1:** Changes in the bOCT catheter compared to the cOCT catheter and rationale for the changes.

Variables	cOCT Catheter	bOCT Catheter	Rationale for the Change
Proximal shaft (high, low profile)	Single-lumen tube	Braided Single-lumen tube	Increases catheter support, increases pushability
Distal shaft	Single-lumen tube	Braided Single-lumen tube, Non-braided single-lumen tube	Integrating the rotating fiber optic core and the movement path of the guidewire reduces the inner diameter, increases pushability, and secures flexibility
Microwire/ guidewire port	Located at the bottom of the distal shaft (hole type)	Located at the top of the distal shaft (skiving mode)	Increases the force supporting the guidewire and minimizes bending of the tube at the guidewire port location
Tip	Connects to the distal shaft by butt welding	Created using the tipping process at the end of the distal shaft	Prevents blood, etc. from entering by minimizing the clearance at the exit of the guidewire passage

**Table 2:** Descriptions of each part of the brain OCT catheter.

Parts of the bOCT Catheter	Description
PIU (connector)	The part that connects to the medical storage and transmission device
Side-arm luer	The part connected to the 3cc syringe for cleaning the catheter before use
Manifold	The part that connects the catheter body, the PIU connector part, and the side-arm luer
Proximal shaft (braided tube)	Part of the catheter close to the PIU connector
Distal shaft (braided tube)	The portion of the catheter close to the tip
Tip	At the end of the catheter, a precise and slender tip allows for smooth movement through tortuous internal structures

**Table 3:** Processing methods for each part of the brain OCT catheter.

Parts of the bOCT Catheter	Processing Method
PIU (connector), manifold	NC (numeric control) milling
Braided proximal shaft (high profile and low profile)	Extrusion, braiding, lamination
Braided distal shaft	Extrusion, braiding, lamination
Non-braided distal shaft	Extrusion
Tip	Tipping
Guidewire port	Skiving
Proximal shaft (high profile) and proximal shaft (low profile) connection	Butt welding, tapering
Catheter shaft, PIU, and side-arm luer connector	Bonding

## Data Availability

Data are contained within the article.

## Supplementary Materials

The following supporting information can be downloaded at: https://www.mdpi.com/article/10.3390/bios15030170/s1. Supplementary Data S1. Description of process method for each part of brain OCT catheter. Supplementary Data S2. Evaluation of the mechanical performance of the catheter.
