# Supplementary material for: Development of a Brain Catheter for Optical Coherence Tomography in Advanced Cerebrovascular Diagnostics
Source: Biosensors (Basel). 2025 Mar 6;15(3):170. doi: 10.3390/bios15030170 (PMC11939904; doi:10.3390/bios15030170)
Supplement: Supplementary file 1 [file biosensors-15-00170-s001.zip › biosensors-3459653-supplementary.pdf]

Supplementary Data S1. Descriptions of the processing methods for each part of the brain OCT catheter.

1) PIU, manifold

Processing method: NC (numeric control) milling

The PIU is an accessory connecting the medical storage and transmission device, while the manifold links the OCT catheter body to the PIU. One of these parts is fabricated using the NC milling process, which automatically cuts materials based on CAD data. For the other, the existing PIU was used for the cerebrovascular OCT catheter by dissolving and utilizing the section where the proximal shaft of the OCT catheter was bonded.

Compared to the cerebrovascular OCT catheter, the connection between the manifold and the PIU in the cardiovascular OCT catheter employs a risk-free luer lock method that locks the manifold in place, preventing it from being dislodged once inserted into the PIU.

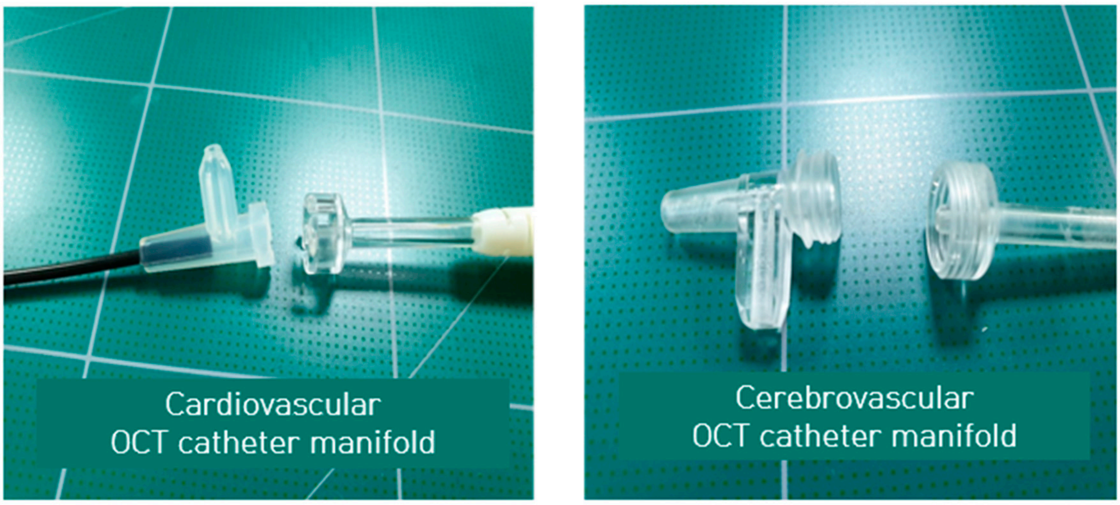

Figure S1. Difference in shape between the cardiovascular OCT catheter manifold and the cerebrovascular OCT catheter manifold.

2) Proximal shaft, distal shaft

- Processing method: extrusion, braiding, lamination

Medical tubes can be classified into non-braided tubes, made solely through the extrusion process, and braided tubes, which involve additional braiding and lamination processes. The catheter made of a braided tube has a braided wire inserted in the tube, so the axial strength of supporting the tube is higher than that of a catheter made of non-braided tubes. This design improves pushability and reduces bending. In cardiovascular OCT catheters, the proximal shaft, which guides the rotating fiber optic core, is typically a non-braided tube. However, cerebrovascular structures are more complex than cardiovascular structures, requiring greater support during procedures. This complexity often leads to weaker pushability and difficulty in

advancing the catheter to the desired position. To address these challenges, a braided tube fabrication method was applied to both the high-profile and low-profile proximal shafts of cerebrovascular OCT catheters, improving strength, pushability, and positioning precision.

The distal shaft of the cerebrovascular OCT catheter is designed to enhance the trackability of the microtubule by incorporating softer materials toward the tip. This reduces guidewire bending and improves the force supporting the tube. A combination of braided and non-braided tubes is utilized for the distal shaft; braided tubes provide structural advantages but cannot be used for tomography because the braided wire obstructs the rotating fiber optic core. For such cases, a skiving process is employed to produce the braided tube.

The manufacturing of both the proximal and distal shafts begins with the extrusion process, which continuously forms products with a uniform cross-section. In this process, a polymer melts in the screw extruder, moves along the screw channel, and is extruded through a mold plate at high pressure. The shape of the mold plate is crucial and varies based on the tube's design and material. Proper mold design requires optimal shape determination, achieved through numerical analysis.

To optimize the extrusion process, the physical and viscoelastic properties of the polymer resin are analyzed, as melted polymers behave as non-Newtonian fluids. Viscosity and shear stress analyses based on shear rates are conducted to understand polymer characteristics. Using these insights, extrusion tools are designed and validated through flow analysis. The final extrusion mold is confirmed by predicting the pressure and flow rate changes at the mold outlet.

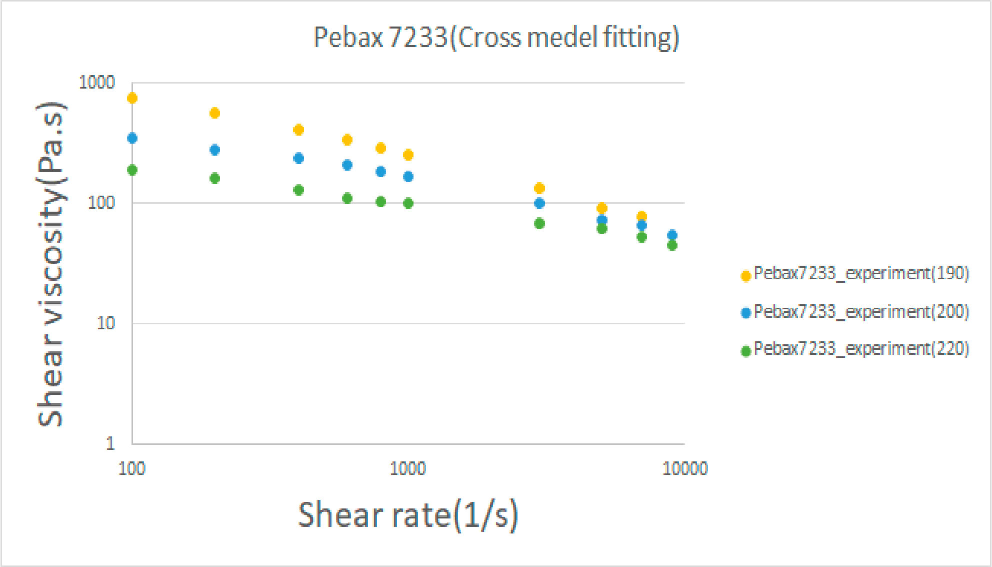

Figure S2. Analysis of viscosity changes based on the temperature and shear rate of the applied polymer resin.

In particular, to minimize defects such as die swelling or shark skin during the extrusion process, numerical analysis is conducted, focusing on the drawdown ratio and land length. By optimizing these parameters and reducing pressure and flow rate deviations, stable tube

extrusion is achieved. Then, the optimal mold (tip and bushing) specification was determined.

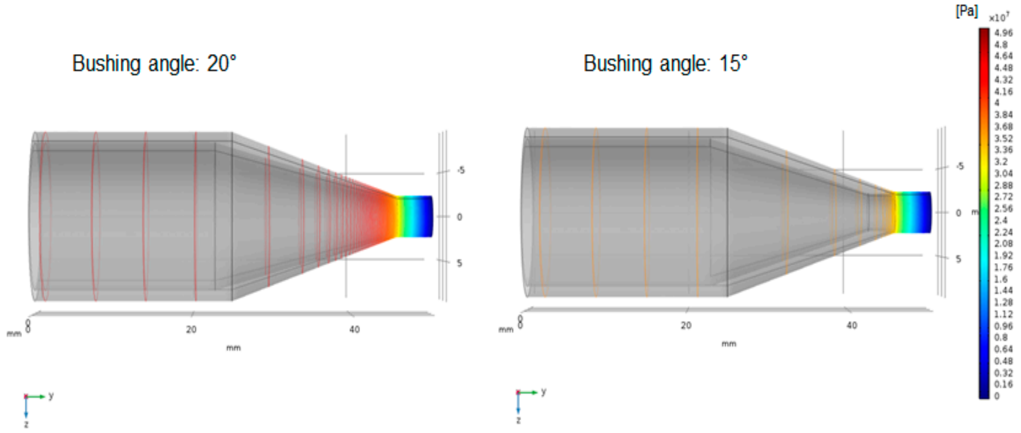

Figure S3. Analysis of the pressure change based on the angle of the bushing in the mold.

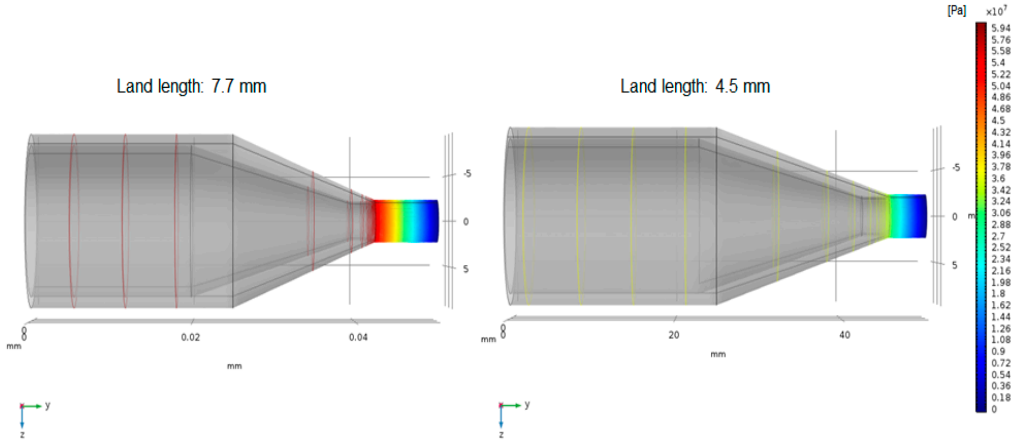

Figure S4. Analysis of the pressure change based on the length of the land in the mold.

Based on the physical properties and numerical analysis results of the polymer resin, the extruder head and mold (tip and bushing) were designed to optimize the flow path of the molten resin. A single-lumen tube was created for the proximal part, while a two-lumen tube was produced for the distal part to design each mold and develop the extrusion process because the material hardness and lumen shape were different.

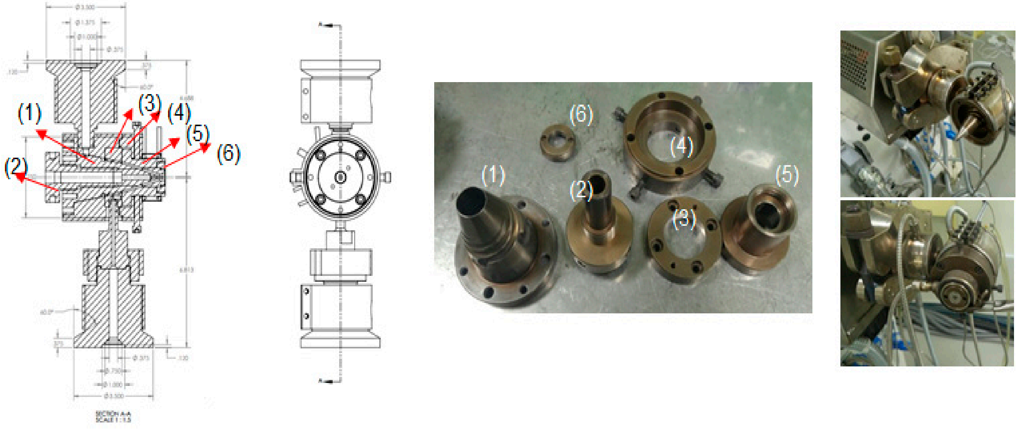

Figure S5. Flow path design/manufacturing and fastening verification of the extruder head part.

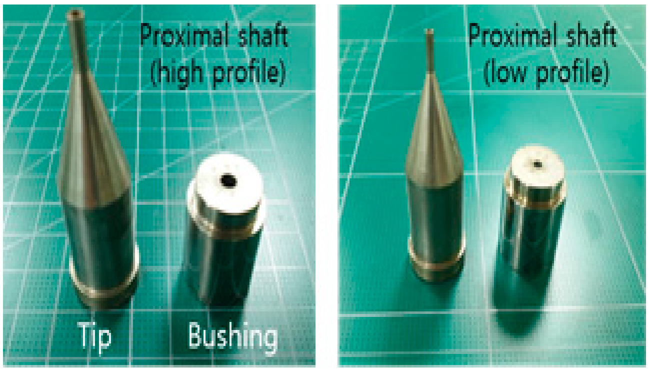

Figure S6. Extrusion mold (tip and bushing) manufacturing based on the numerical analysis results.

Therefore, an optimal extrusion process was developed by aligning material properties and tube design with key parameters like melting temperature, air injection velocity, screw and puller speed, and vacuum degree to ensure precise and efficient tube production.

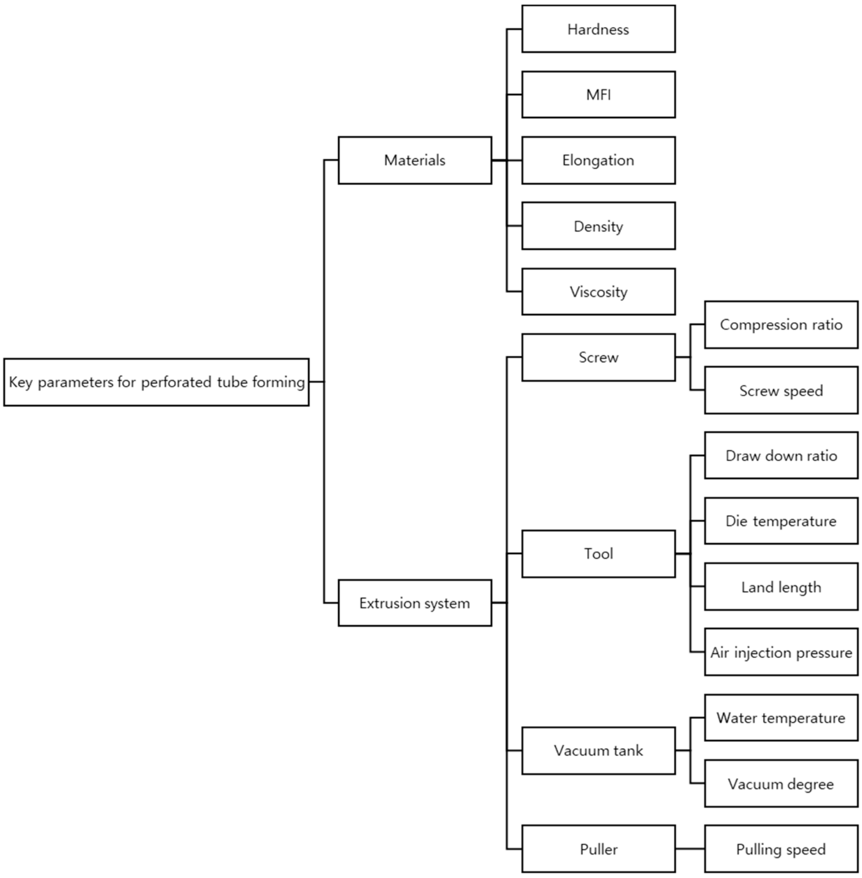

Figure S7. Key parameters for perforated tube forming.

To produce a braided tube, the PTFE liner, the braided wire, and the outer jacket must be layered sequentially, followed by a lamination process where the layers are fused using high-temperature air.

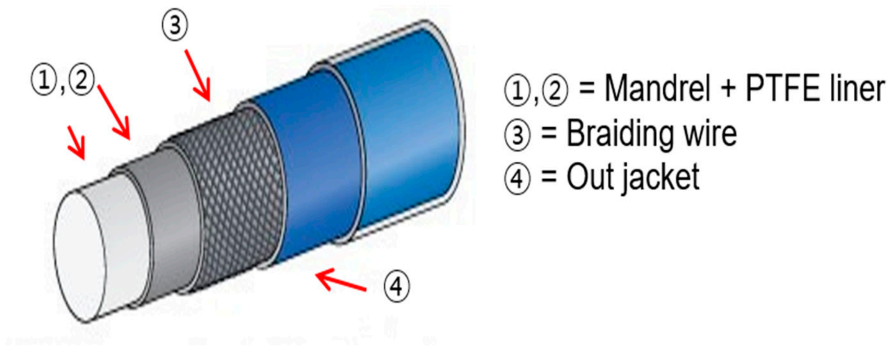

Figure S8. Braided tube configuration.

While making a braided tube, the outer jacket is created first. This requires careful selection of the tube material, inner diameter (ID), and outer diameter (OD). A medical polymer with a hardness of 70D was used for the material. The ID of the outer jacket was designed to be larger than the OD of the laminated tube to allow easy insertion of the PTFE liner and braided wire assembly. The OD was determined based on the required wall thickness of the tube.

The dimensions of the proximal shaft (high profile) of the cerebrovascular OCT catheter were selected by referencing the proximal shaft (high profile) dimensions of the cardiovascular OCT catheter. The cardiovascular OCT catheter's proximal shaft has an OD of 2.18 mm, an ID of 1.57 mm, and a wall thickness of 0.30 mm. For the outer jacket of the cerebrovascular OCT catheter, the ID and OD were determined based on the dimensions of the laminated tube layers as follows:

PTFE liner ID/OD: 1.27 mm / 1.34 mm

Braiding wire thickness: 200 μm

Laminated tube (PTFE liner + braiding wire) ID/OD: 1.27 mm / 1.44 mm

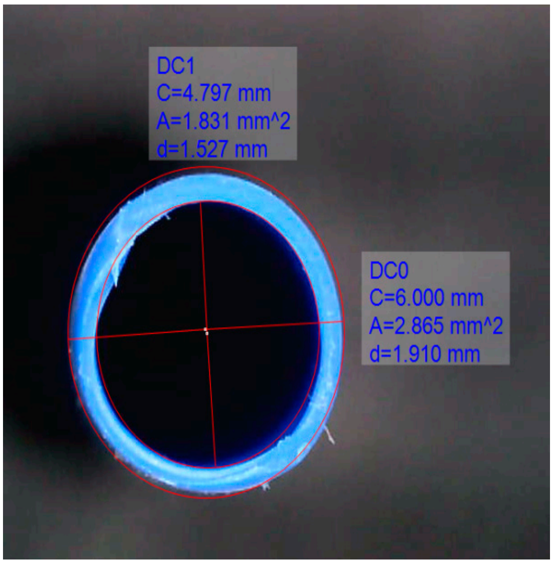

Figure S9. Outer jacket for a braided proximal shaft (high profile) tube.

Considering that the maximum diameter of the rotating fiber optic core inserted into the proximal shaft (high profile) is 1 mm, a PTFE liner with an inner diameter of 1.27 mm and an outer diameter of 1.34 mm was selected. If the braiding wire is laminated on the PTFE liner, a tube with an outer diameter of 1.44 mm and a thickness of 130 μm is completed. The inner diameter of the outer jacket was selected as 1.7 mm, which is about 260 μm larger than the outer diameter of the tube, for easy insertion into the stacked tube. Considering that the existing proximal shaft (high profile) tube thickness is 0.15 mm, in order to complete the final wall thickness close to 300 μm, including the wall thickness of the stacked tube (125 μm), the outer diameter with a wall thickness of 150 μm was selected as 1.92 mm.

As shown in Figure S9, the final outer jacket with an outer diameter of 1.91 mm, an inner diameter of 1.7 mm, and an ovality of 0.036 was completed. There is a 10 μm difference between the outer diameter of the finished outer jacket and the selected outer jacket, but it is considered to be within the tolerance range.

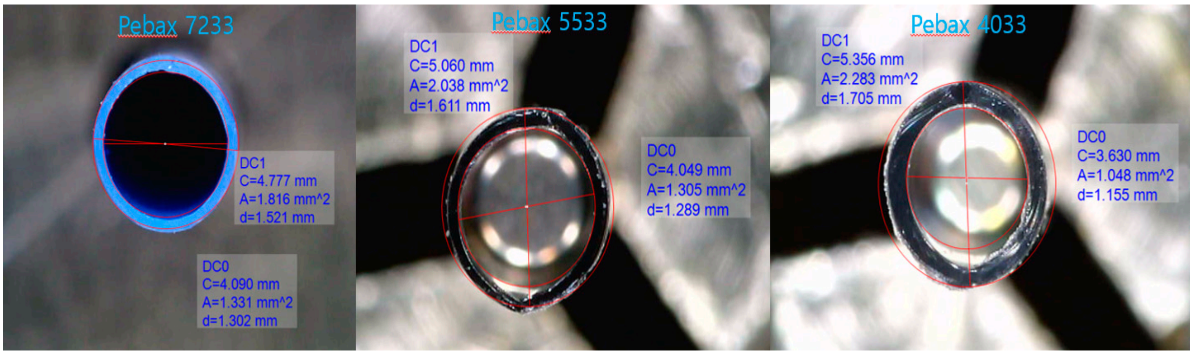

Figure S10. Outer jacket for a braided distal shaft (high profile) tube.

The inner and outer diameters of the cerebrovascular OCT catheter's proximal shaft (low profile) were selected as follows:

PTFE liner inner/outer diameter: 1.0 mm / 1.11 mm

Braiding wire: 100 μm

PTFE liner + braiding wire inner/outer diameter: 1.0 mm / 1.17 mm

Considering that the minimum diameter of the rotating fiber optic core inserted into the proximal shaft (high profile) is 0.4 mm and the guidewire diameter is 0.35 mm, a PTFE liner with an inner diameter of 1.0 mm and an outer diameter of 1.11 mm was selected. When stacking the braiding wire over the PTFE liner tube of an outer diameter of 1.17 mm, a thickness of 85 μm is produced. The inner diameter of the outer jack was selected as 1.3 mm, which is about 100 μm larger than the outer diameter of the tube, for easy insertion into the stacked tube. Since the thickness of the existing proximal shaft (high profile) tube is too thin at 0.0725 mm, the final wall thickness, including the wall thickness (125 μm) of the tube stacked in the same way as the braided proximal shaft (high profile), was designed close to 300 μm. Therefore, a wall thickness of 200 μm and an outer diameter of 1.5 mm were selected. Pebax 4033 is a non-braided shaft designed with an inner diameter of 1.1 mm.

During the braiding process, the core of the braided tube was analyzed to determine an appropriate braiding angle by measuring tensile strength and bending strength (buckling) at various angles. Based on these results, the braiding conditions were finalized, with the selected braiding angle set at 45 PPI.

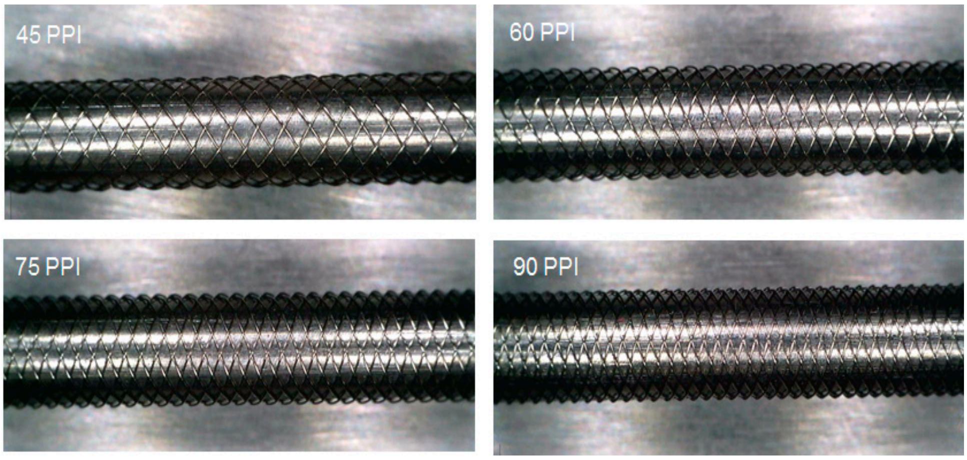

Figure S11. Development of the braiding process for each PPI.

The proximal shaft (high profile) of the cerebrovascular OCT catheter underwent the lamination process where an extruded tube and braided wire bundle were fused using hot air. A single-lumen braided tube with an outer diameter of 5.0 Fr was successfully developed by adjusting the temperature of the injected air to 200 °C and using a scanning speed of 1.0 mm/s.

The ovality of the tube was measured using the following equation:

$$\text{Ovality: } 2 \times (\text{Dmax} - \text{Dmin}) / (\text{Dmax} + \text{Dmin}),$$

where Dmax is the maximum tube outer diameter (1.69 mm) and Dmin—minimum tube outer diameter (1.67 mm): 2 × (1.69 – 1.67) / (1.69 + 1.67) = 0.0119

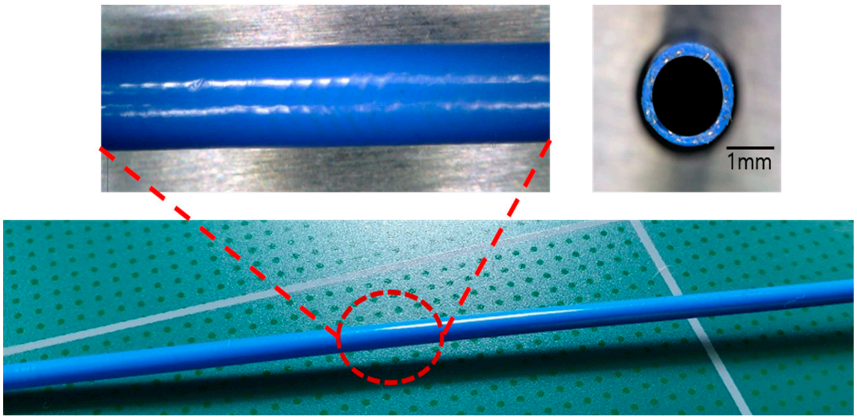

Figure S12. Single-lumen braided tube (45 PPI) of the proximal shaft (high profile).

The proximal shaft (low profile) of the cerebrovascular OCT catheter also completed the lamination process under the same conditions as the proximal shaft (high profile) to develop a single-lumen braided tube with an outer diameter of 3.9 Fr as follows.

In the case of ovality, it was measured using the following equation:

$$\text{Ovality: } 2 \times (\text{Dmax} - \text{Dmin}) / (\text{Dmax} + \text{Dmin}),$$

where Dmax is the maximum tube outer diameter (1.32 mm) and Dmin—minimum tube outer diameter (1.28 mm): 2 × (1.32 – 1.28) / (1.32 + 1.28) = 0.03

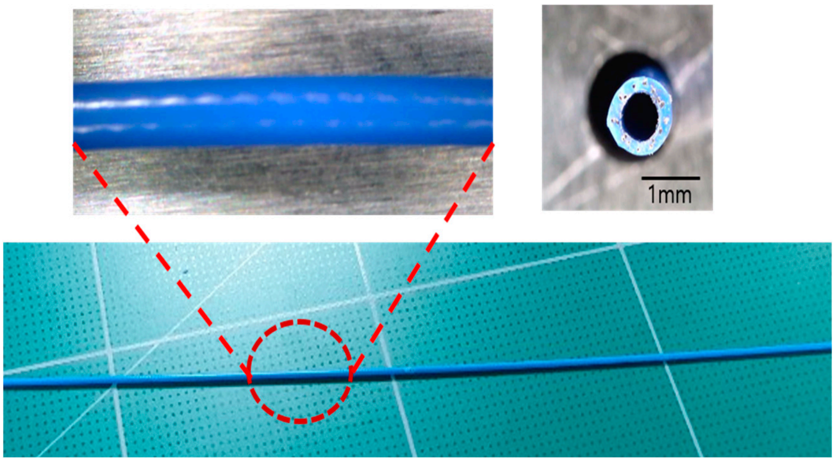

Figure S13. Single-lumen braided tube (45 PPI) of the proximal shaft (low profile).

3) Tip

Processing method: tipping

The tip of the cerebrovascular OCT catheter is located at the end of the distal shaft, helping it move smoothly through complex internal structures and acting as the exit for the guidewire inserted through the guidewire port. It is made using a tipping process on a multifunctional catheter manufacturing platform, which can perform such tasks as tapering, tipping, butt welding, flaring, and closed lumen processes. In the tipping process, the tube is placed in a specially designed jig and heated with a high-frequency heater while being simultaneously pushed into the jig. This dual-action process deforms the tube to match the inner shape of the jig. Once shaped, the tip is cooled using a cooling system, completing the final shape of the tip.

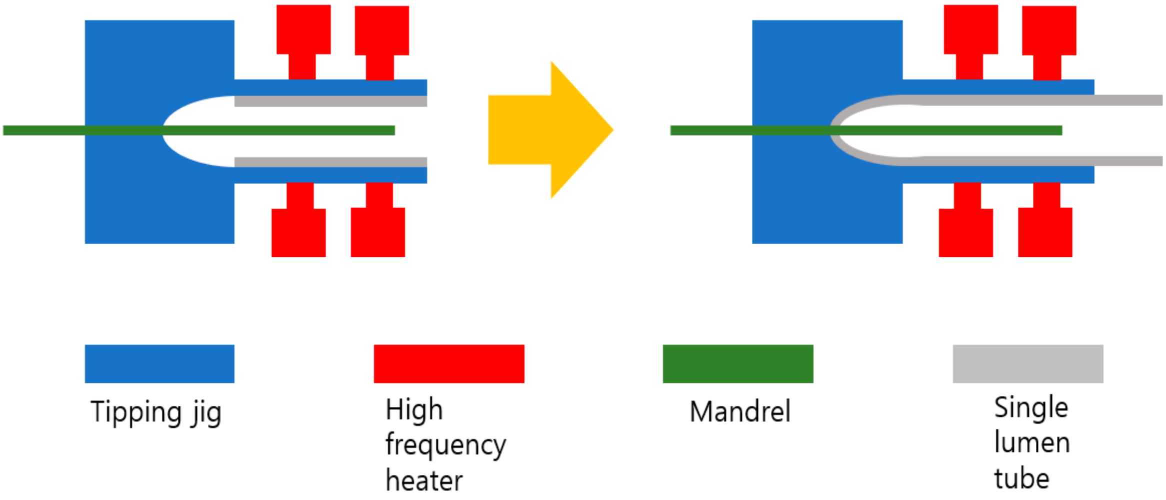

Figure S14. Schematic diagram of the tipping process.

To complete the tip of the cerebrovascular OCT catheter, a specially designed jig is needed to match the curved shape of the tip. The tip has a curvature at the end and a reduced lumen diameter, allowing only the guidewire to exit while maintaining a passage for the rotating fiber optic core. The internal structure of the tipping jig is built to accommodate the tip's curvature and includes a mandrel to shape the tip accurately. The tipping process begins by fastening the completed jig to a multipurpose machine. The distal shaft's end is then inserted into the jig, ensuring the lumen, which serves as the guidewire passage, is properly aligned with the mandrel. The next step involves determining the tube's insertion length (tip length) into the jig and setting the force required to push the tube. The multipurpose machine is equipped with controls for adjusting both the tip length and the pushing force. A rod and screw mechanism beneath the high-frequency heater manages the tip length. The rod defines the insertion point at the jig frame, while the distance from the rod to the screw determines the tip's length. Additionally, a force adjustment device on the machine allows the pushing force to be set on a scale from 1 (lowest) to 6 (highest). To produce a straight, non-bent tip, minimal pushing force should be applied.

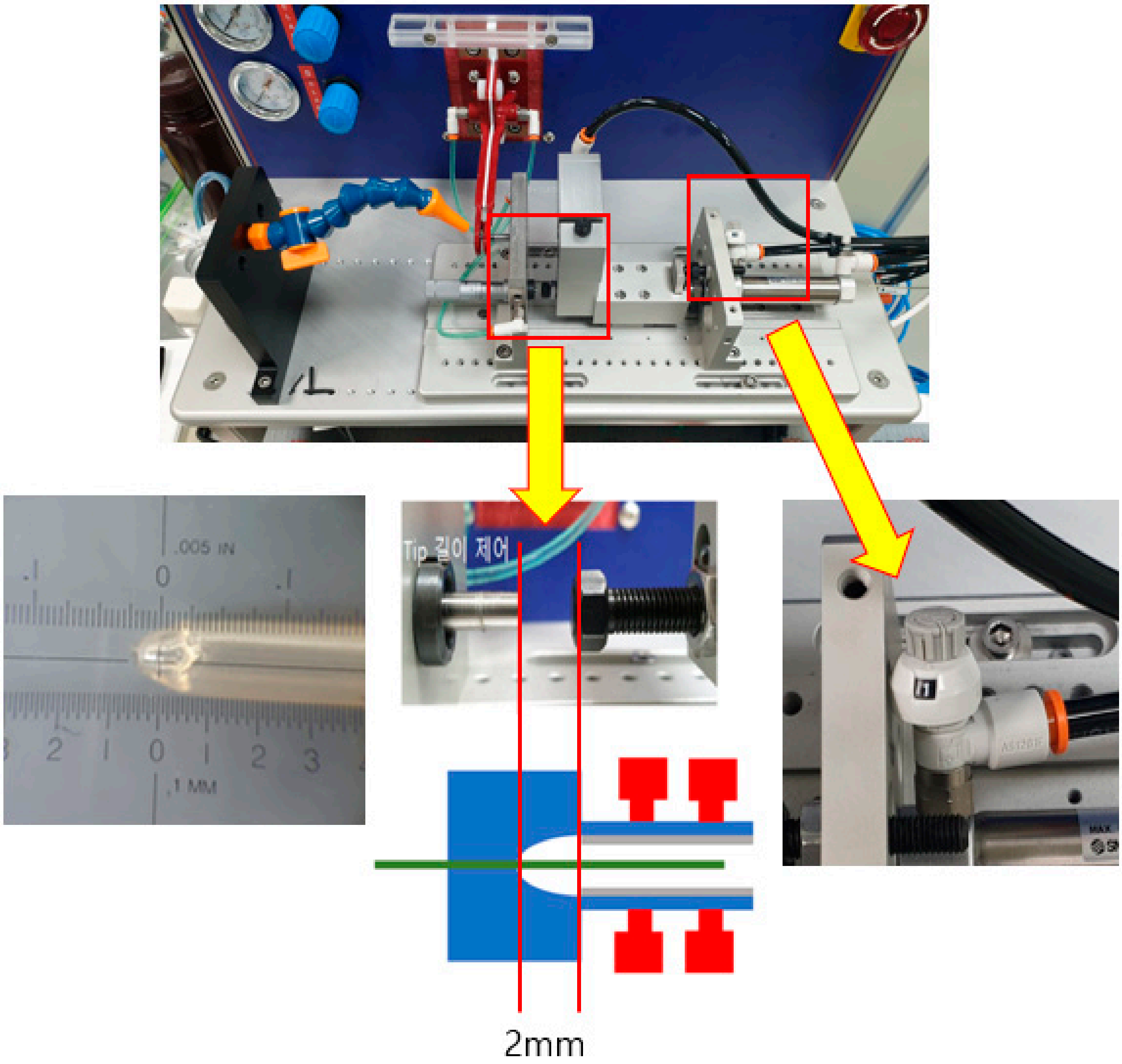

Figure S15. Insertion length and pushing force based on the length of the tip.

For the cerebrovascular OCT catheter, the tip length was set to 1 mm, and the length from the rod to the screw was set to 2 mm. This 2 mm setting was chosen to allow for a larger amount of melting due to the reduced lumen size. To ensure the tip remained straight and non-bent, the force control device was set to 1, applying minimal force to push the tube during the process.

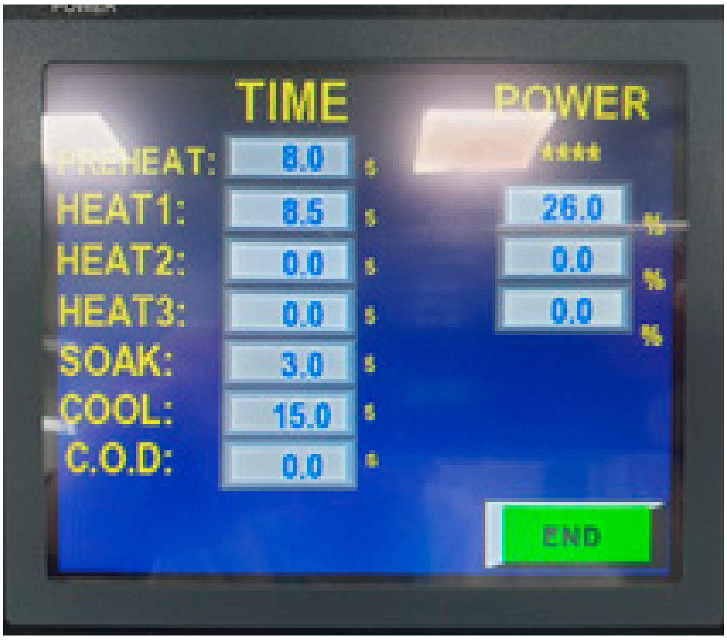

Figure S16. Time and power settings.

In a multipurpose machine, setting the time and power is critical for achieving the desired final shape. The application of heat for a few seconds, combined with a specific power percentage, determines the outcome. Power can be adjusted between 1% and 100%, with typical values set between 10% and 30%. Higher power levels generate more heat, and settings above 30% risk melting the tube inside the jig. Pre-heat is the duration (in seconds) that the heat is applied before the tube is pushed into the jig at the set power level. Heat1 refers to the heating duration after the tube is fully inserted into the jig. The actual heating time inside the jig is calculated as Heat1 – Pre-heat. Typically, Heat1 is set slightly higher than Pre-heat, usually by 0.5 to 1 second. Cool represents the time high-pressure air is applied at the end of Heat1. Achieving the desired tip shape requires numerous experiments to optimize these settings based on the tube material and final shape requirements. For the cerebrovascular OCT catheter tip, the optimal conditions were determined to be 26% power**,** 8.0 seconds Pre-heat**,** and 8.5 seconds Heat1, with the cooling time fixed at 15 seconds.

4) Guidewire port

Processing method: skiving

The guidewire port, the entry point for inserting the guidewire, is commonly created using either the drilling or skiving process. Traditional cardiovascular OCT catheters typically use the drilling process, with the guidewire port located on the lower part of the distal shaft. However, this placement reduces support for the guidewire, increasing the risk of kinking during insertion. In cerebrovascular OCT catheters, the design prioritizes better guidewire support and enhanced catheter control. To achieve this, the guidewire port is positioned at the upper part of the distal shaft, closer to the operator, and is created using the skiving process.

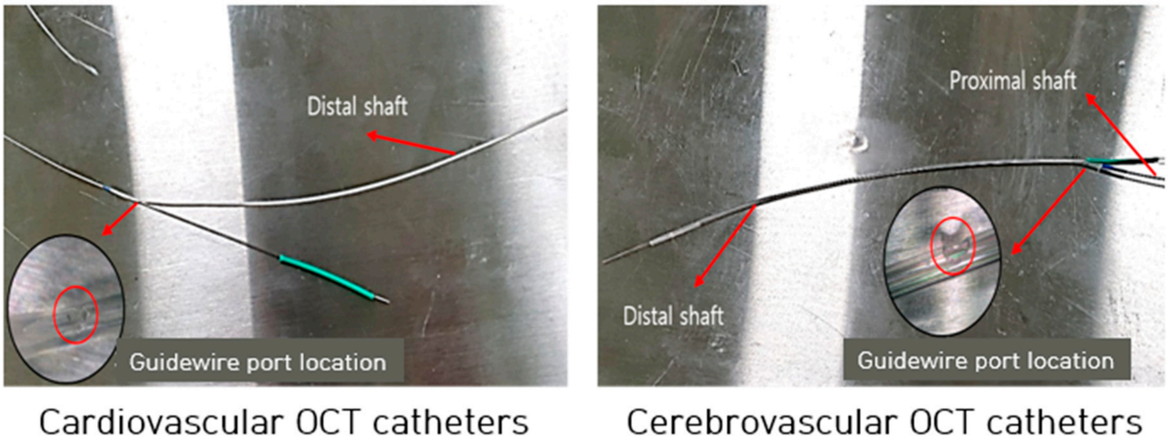

Figure S17. Guidewire port location for cardiovascular OCT catheters and cerebrovascular OCT catheters.

When the guidewire port is relocated from the bottom to the top of the distal shaft using the drilling process, the insertion angle between the guidewire port and the guidewire becomes 90°. This change increases the distance to the catheter tip, leading to greater friction during guidewire insertion. To minimize this friction, the skiving process is used instead, as it creates a smaller insertion angle compared to the drilling process.

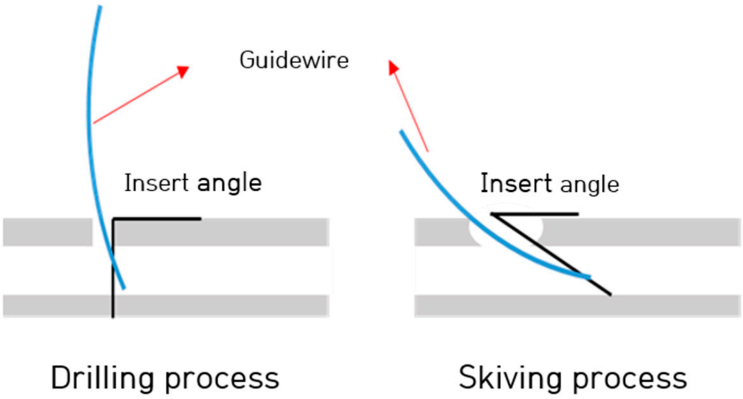

Figure S18. Insertion angle difference between the drilling process and the skiving process.

The skiving process utilizes the same medical drilling machine as the drilling process, but differs in its use of a skiving punch and a catheter guide. Unlike the drilling process, it does not require a mandrel or hole plug ejection pins.

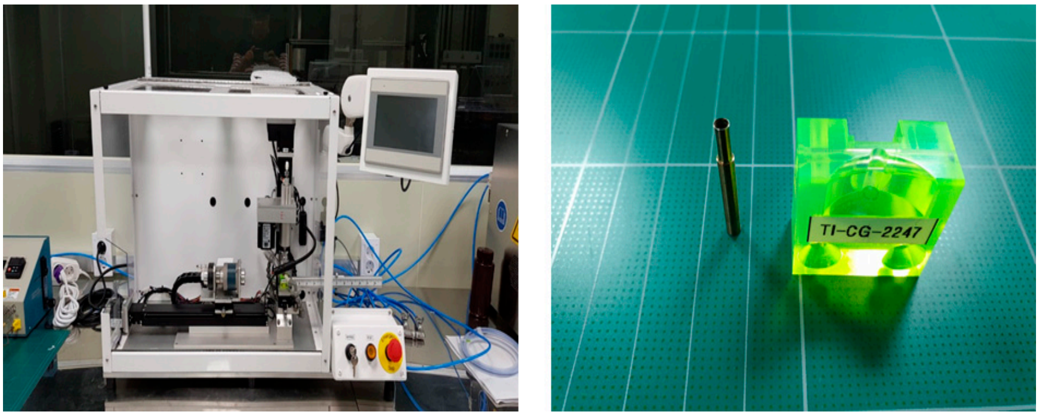

Figure S19. A medical drilling machine, a skiving punch, and a catheter guide.

The distal shaft material, with a softness of approximately 50D, requires a high spindle speed for precise skiving. When skiving softer tubes, a low spindle speed can result in incomplete cuts and a rough surface. To ensure a clean cut, the spindle speed was set at 1500 rpm. For harder materials with a hardness of 60–70D, the recommended spindle speed is typically 1000–1200 rpm.

Regarding tool depth, the cutting depth varies based on the tube's diameter: larger diameters require deeper cuts, while smaller diameters require shallower ones. To avoid frequent adjustments for different tube sizes, a fixed tool depth of 0.15", the maximum depth the punch can achieve along the z-axis, was chosen.

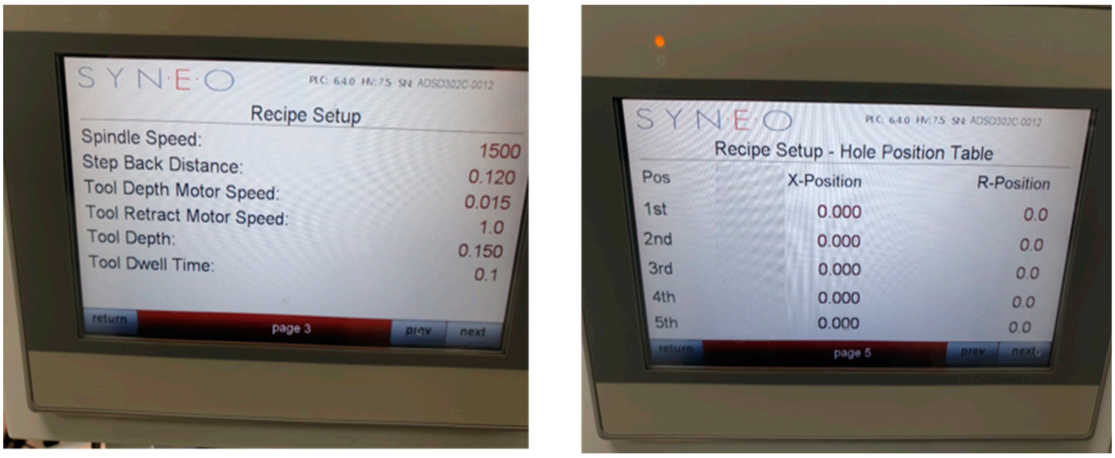

Figure S20. Medical drilling machine setup for skiving.

For the cerebrovascular OCT catheter, skiving was performed only once, so the default values (x-position: 0, R-position: 0) were used.

To ensure precision, the tube section to be skived and the punch must be perfectly perpendicular. Since the skived area is a small hole for the guidewire, the tube must be inserted into the catheter guide to align the hole vertically with the punch.

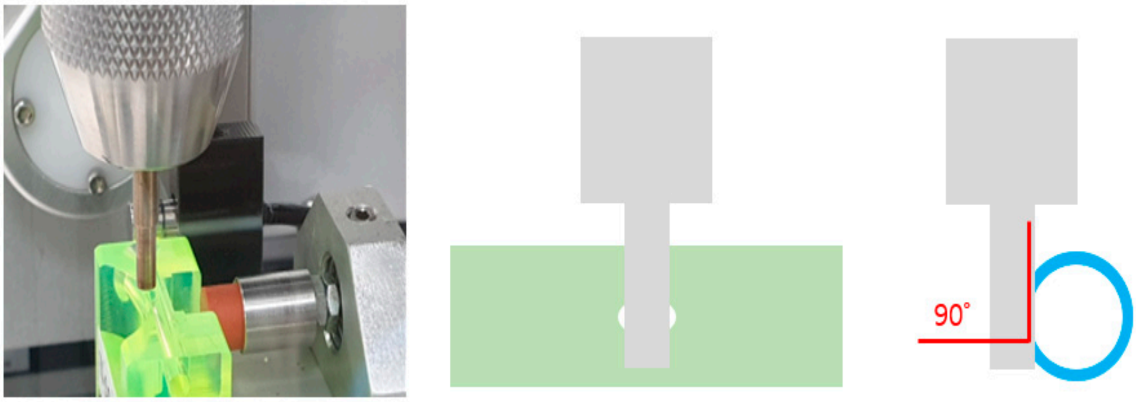

Figure S21. Final schematic diagram of skiving.

Finally, the skiving process was carried out with the spindle speed set to 1500 rpm, tool depth at 0.15", and the recipe setup-hole position table using default values (x-position: 0, R-position: 0). After aligning the punch and the small hole vertically, the skiving process was performed, resulting in a clean cut for the guidewire port, as shown below.

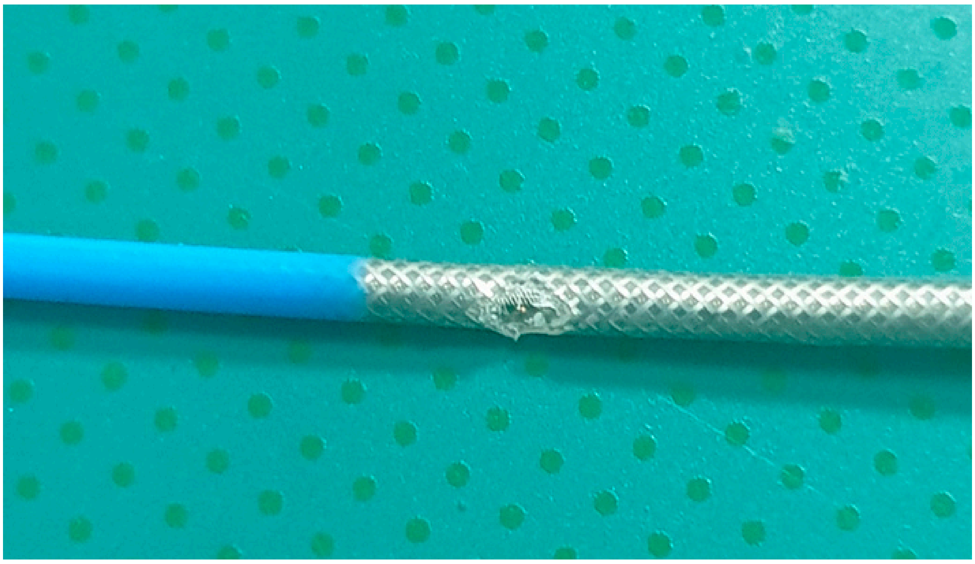

Figure S22. Guidewire port.

5) Proximal shaft (high profile) and proximal shaft (low profile) connection

- Processing method: tapering, butt welding

The connection between the proximal shaft (high profile) and the proximal shaft (low profile) serves as the transition point between these two sections. In cardiovascular OCT catheters, tapering is performed from the end of the high-profile proximal shaft to the start of the low-profile proximal shaft. In order to make the same shape for the cerebrovascular OCT catheter, tapering and butt welding processes were adopted.

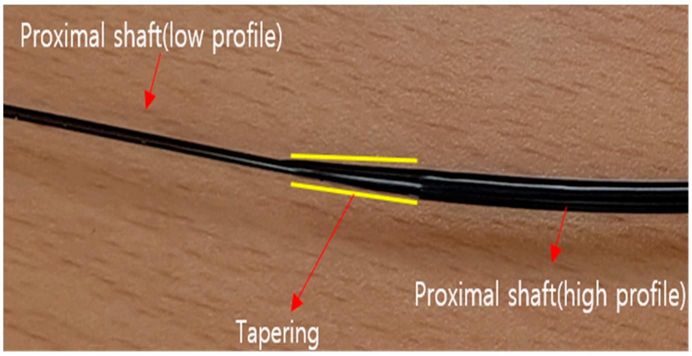

Figure S23. Cardiovascular OCT catheter's proximal shaft's connection shape.

Two processes were used to perform tapering and butt welding. A taper mandrel was used, which served as a frame for shaping the outer tube into a taper. For butt welding, heat-applied equipment was used to connect the different tubes, with parameters such as power, soak time, drive force, and cooling time carefully optimized.

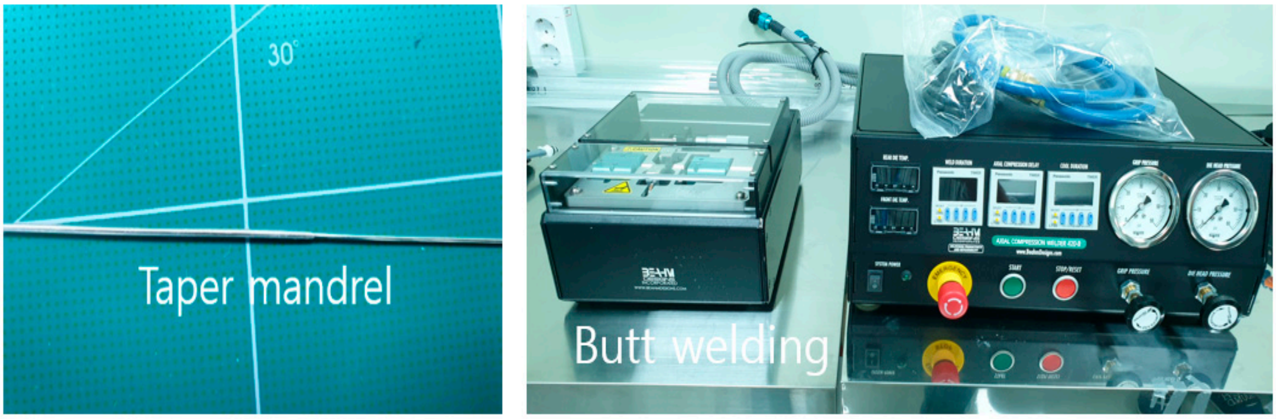

Figure S24. Equipment required for the butt welding and tapering processes.

The proximal shaft of the cerebrovascular OCT catheter was designed as a braided tube to reduce tube bending and improve pushability during procedures. However, shaping the braided tube into a taper and welding tubes of different diameters added complexity to the process. To address this, a heat-deformable single-lumen tube was used. A taper mandrel, combining a large-diameter and a small-diameter mandrel in a taper shape, facilitated the tapering process. The braided proximal shaft (low profile) was inserted into the small-diameter section, while the single-lumen tube was inserted into the large-diameter section.

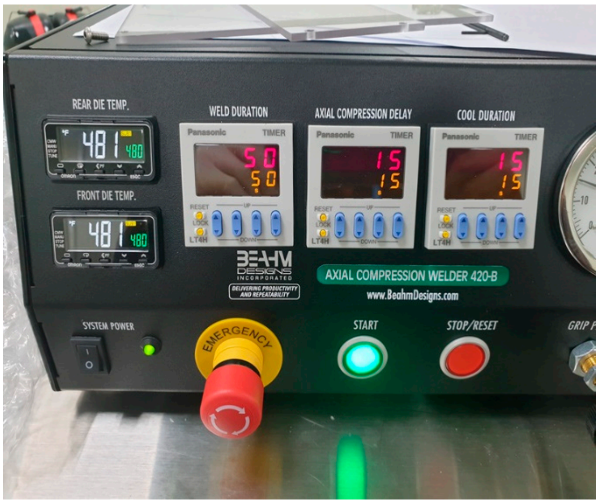

Figure S25. Butt welding settings.

The butt welding machine was set to a temperature of approximately 250 °C (480 °F). The heating time was adjusted to 50 s to ensure that the tube and the shrink tube melted sufficiently and contracted. The pushing process began 15 seconds after heating, followed by a cooling period of 15 seconds to allow the materials to cool sufficiently.

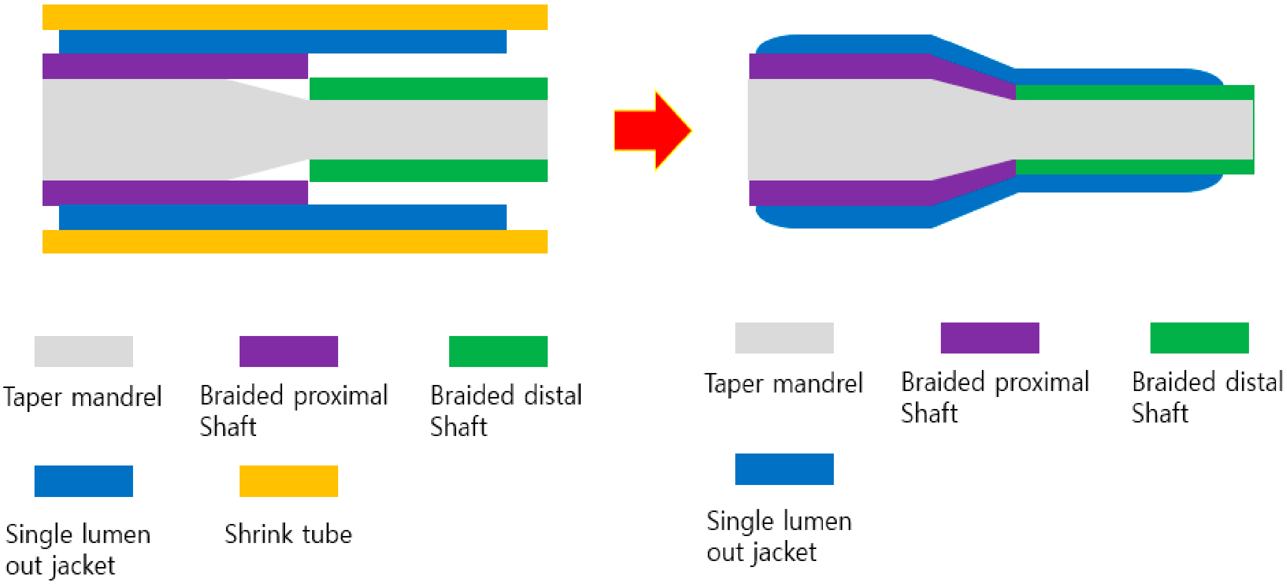

Figure S26. Schematic diagram of the tapering process.

This method minimizes the inner diameter of the catheter to suit the extremely small size of cerebral blood vessels. The large lumen is designed to accommodate the mandrel of the rotating coil, while the small lumen is designed specifically for the rotating coil itself.

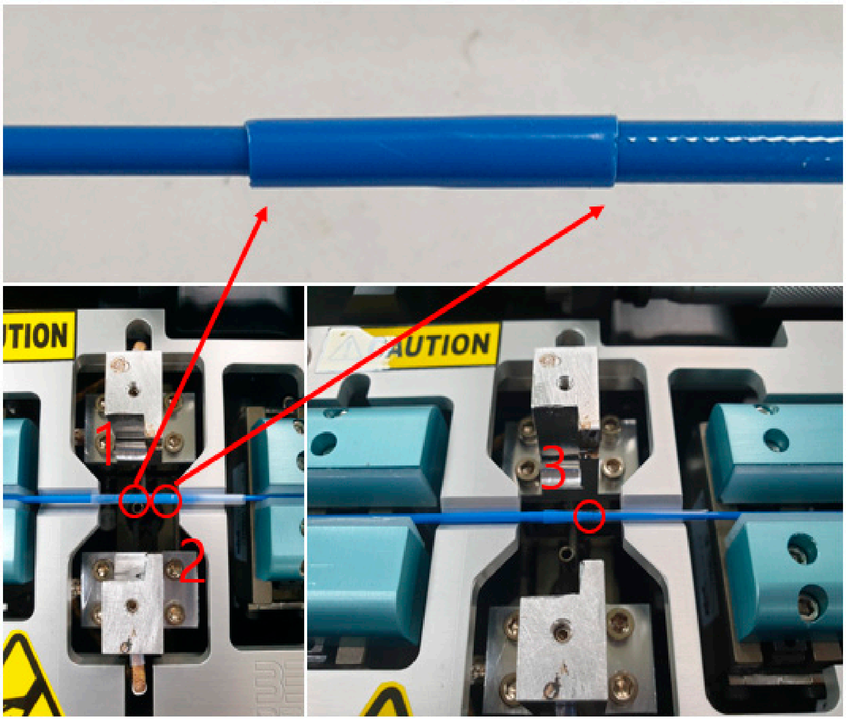

Figure S27. Butt welding process.

In the picture above, butt welding is performed once on each side. After that, part 1 is performed one more time because the small part of the outer diameter shrinks less, leading to burr formation.

Afterward, the heat shrink tube is removed, and the tube is inserted into the taper mandrel. The single-lumen tube and the braided proximal shaft (low profile) are aligned with an overlapping section of 5–10 mm. A longer overlap allows the single-lumen tube to melt from the heat, increasing the joint area with the proximal shaft (low profile) and resulting in a stronger bond. The braided proximal shaft (high profile) and the braided proximal shaft (low profile) were successfully attached in a taper shape completing the butt welding process.

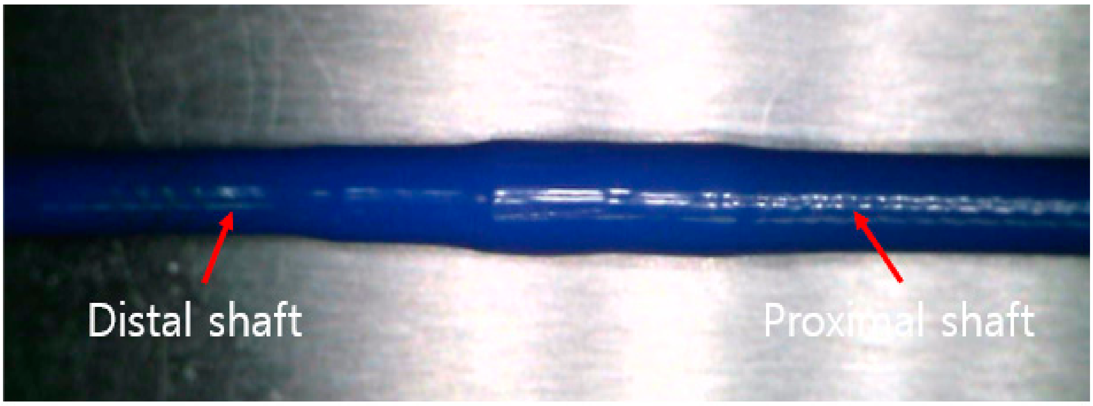

Figure S28. Proximal shaft (high profile) and proximal shaft (low profile) connection.

6) Catheter shaft, PIU, and side-arm luer connector

- Processing method: Bonding

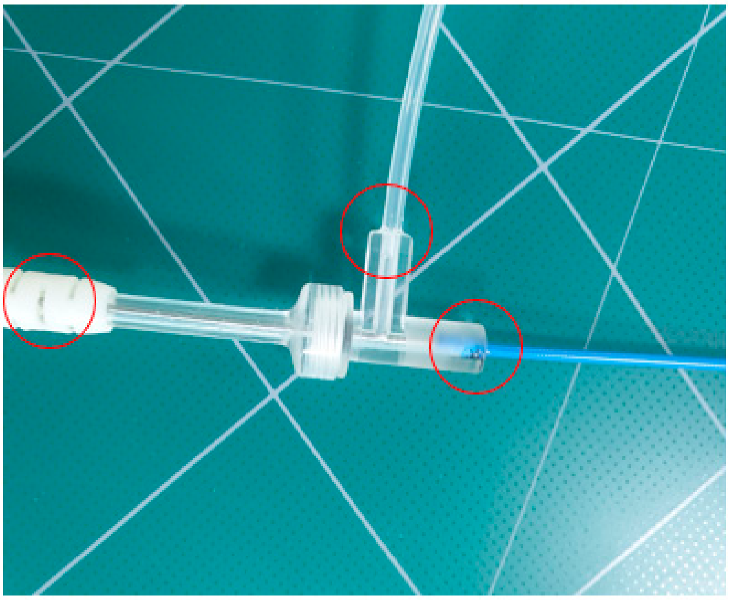

Figure S29. Bonding position in the bonding process.

The catheter shaft, PIU, and side-arm luer are connected as shown in the figure 29. The connection was hardened and securely adhered to the red part through the UV bonding process.

7) Hydrophilic coating

Blood consists of plasma and blood cells, with plasma making up about 55%, and water accounting for 90% of the plasma. To reduce friction during procedures, a hydrophilic coating was applied to the catheter shaft. When saline is continuously applied to the hydrophilic coating during insertion into blood vessels, the friction between the catheter shaft and the blood significantly decreases, greatly enhancing the catheter's pushability.

The hydrophilic coating was applied through dip coating using a hyaluronic acid (HA)-based solution, followed by heat curing. An analysis of the contact angle after coating confirmed that the friction force was reduced, particularly when passing through curved blood vessels.

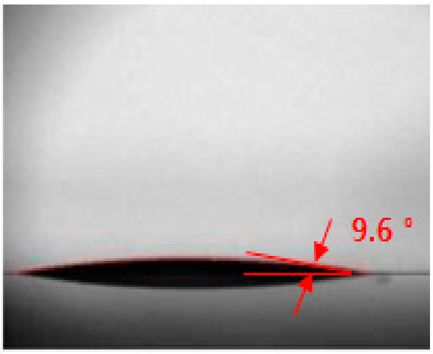

Figure S30. Surface contact angle after hydrophilic coating.

- Reinforcement of the distal tip shape and marker band function

Initially, the catheter tip was 15 mm long, which posed several issues. The excessive length made it difficult for operators to precisely locate the catheter under X-ray imaging and increased the risk of blood vessel damage, especially in highly curved vessels. To address these challenges, the second prototype reduced the tip length to 2 mm and added a marker band containing radiopaque material. These changes improved visibility and reduced the risk of vessel damage, effectively overcoming the drawbacks of the earlier design.

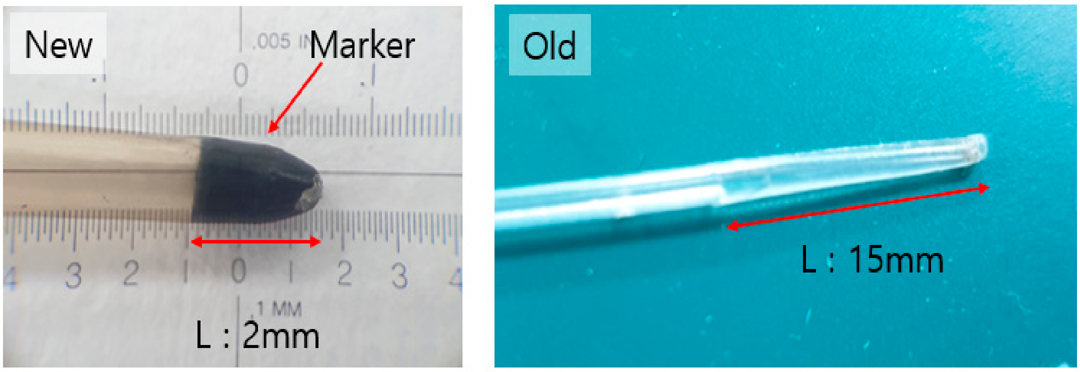

Figure S31. Distal tip, enlarged view.

8) Dual-lumen and single-lumen tubes

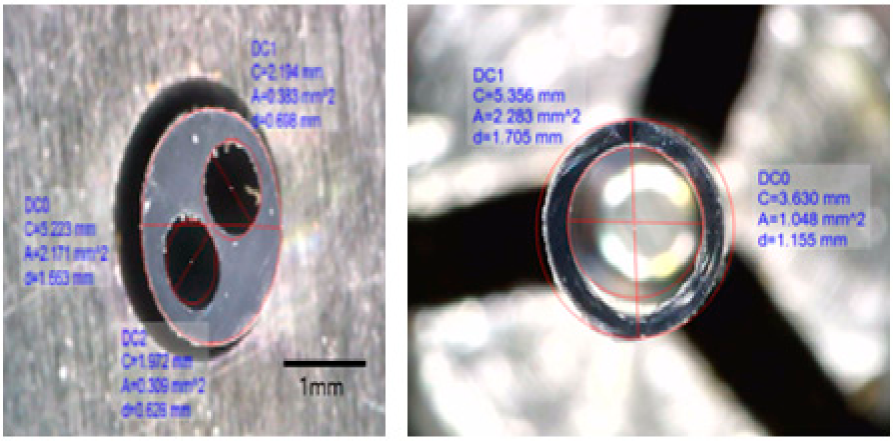

Figure S32. Distal section comparison between dual-lumen and single-lumen tubes.

The existing methods were reviewed to simplify the distal part processing and minimize its diameter. Simplification was achieved by eliminating the hub welding process and transitioning from a double-lumen to a single-lumen design, making the manufacturing process more efficient. To minimize the diameter, the middle wall was removed, resulting in a reduction of approximately 0.2 mm.

9) Guidewire pass length and configuration

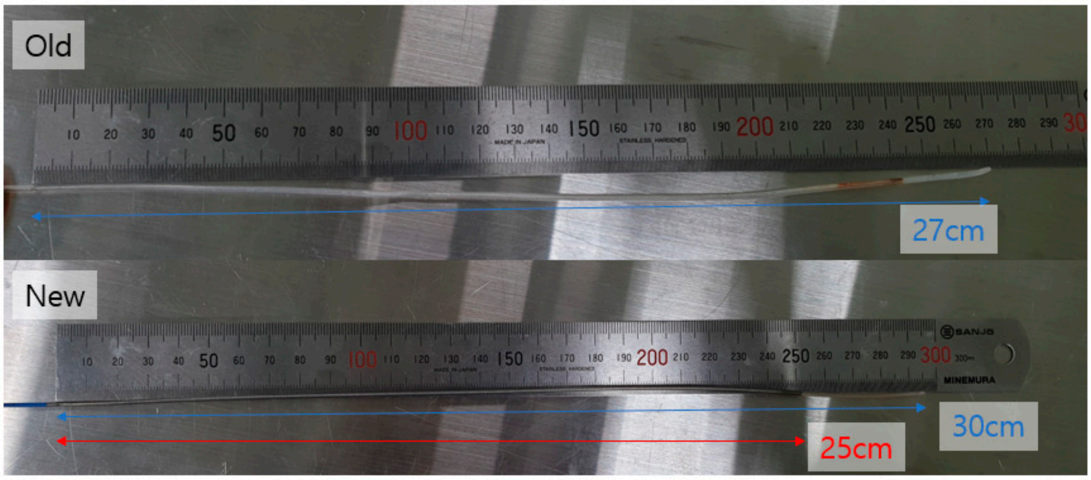

Figure S33. Distal part shape comparison

The initial design of the catheter featured a non-braided distal part with a guidewire passage length of approximately 270 mm. However, this design faced challenges in pushability and trackability, making it difficult to traverse curved passages reliably. To address these issues, the updated catheter features a slightly longer passage length of the guidewire and the braided section extended by about 250 mm, improving pushability. Additionally, to enhance trackability, the tip part (30 mm) was made from a soft material (Pebax 4033) and kept non-braided.

10) Availability of 6 Fr guiding catheters

Cerebrovascular blood vessels are very small, with diameters of less than 2.5 mm, and typically require 7–8 Fr or 6 Fr guiding sheath catheters for a quick access to the lesion. Ensuring a smooth entry of the OCT catheter into a sheath catheter is crucial. To achieve this, the welding process was improved compared to the first prototype, resulting in a more stable entry, as confirmed in the figure below.

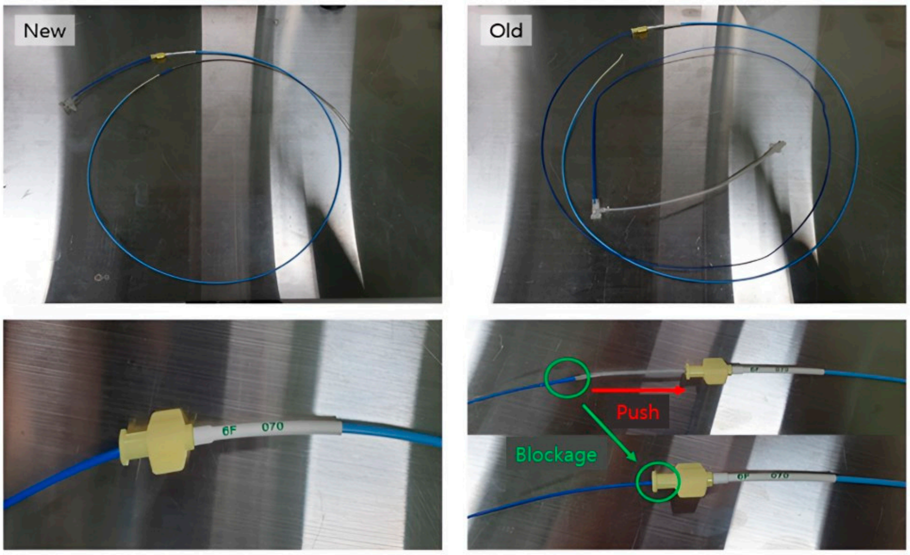

Figure S34. Compatibility test for a 6 Fr Shuttle catheter (ENVOY).

Supplementary Data S2. Evaluation of the mechanical performance of the catheter.

Tube-type medical devices such as catheters, guidewires, stent delivery devices, and endoscopes were tested and evaluated using the ASTM F2394 standard flow path. A motor with a roller was driven to transport the catheter and the force sensor measured the force, and this process was recorded in real time using an image camera.

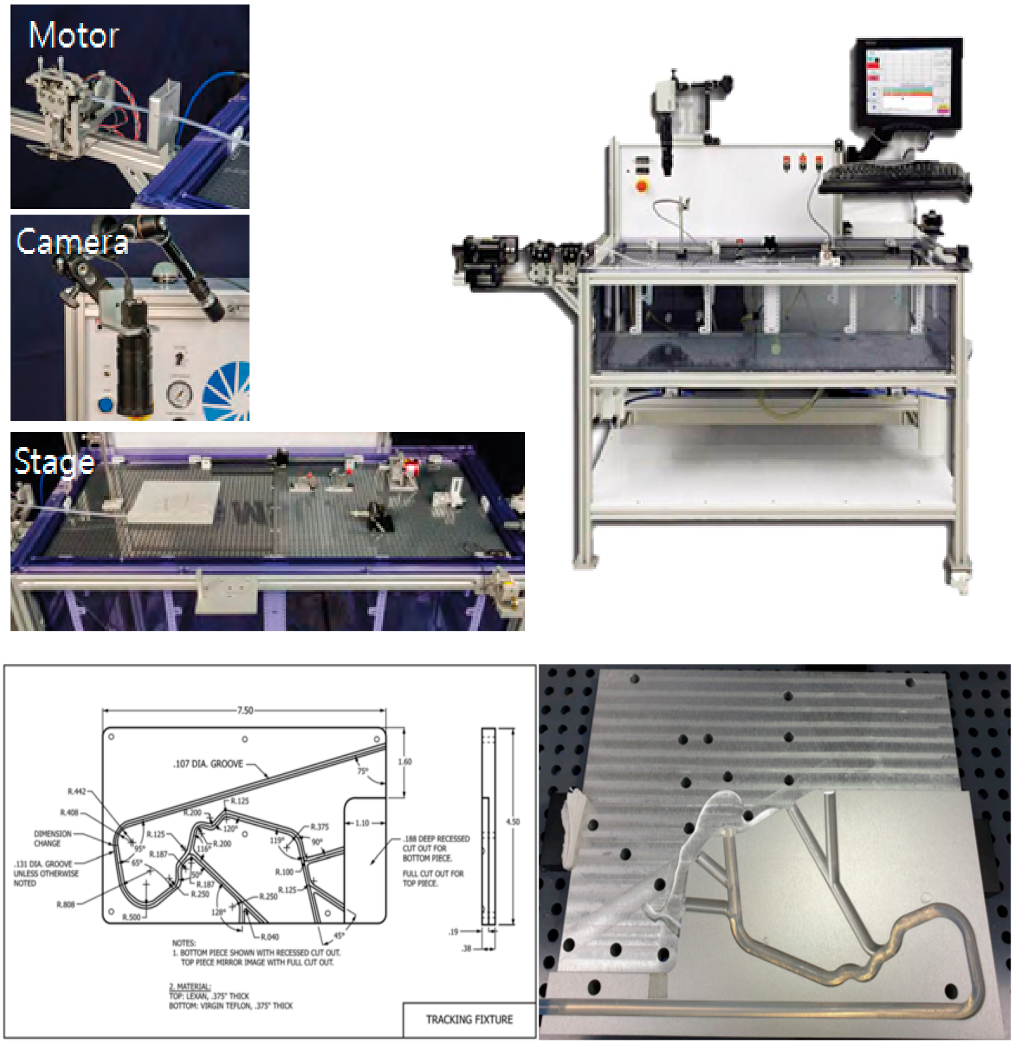

Figure S35. Catheter trackability measuring device and the ASTM F2394 standard flow path.

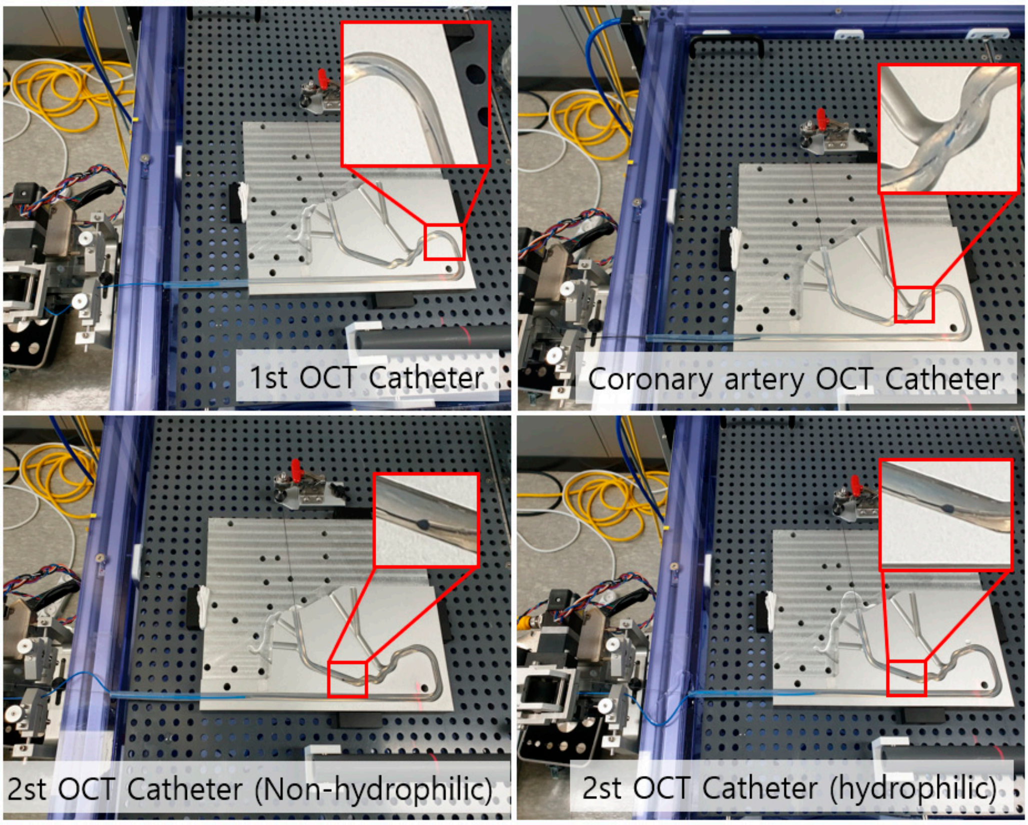

Figure S36. IDTE test experiment method.

In this process, distilled water was used in the flow path instead of blood, and a 0.014-inch guidewire with a fixed tip was employed to prevent movement. The moving distance was set to 240 mm from the laser point, matching the flow path length of approximately 240 mm, to test the catheter up to its kinking point. The kinking point corresponds to the location where the catheter experiences the maximum drag force.
